# Triterpenoidal Saponins from the Leaves of *Aster koraiensis* Offer Inhibitory Activities against SARS-CoV-2

**DOI:** 10.3390/plants13020303

**Published:** 2024-01-19

**Authors:** Ji-Young Kim, Tai Young Kim, So-Ri Son, Suyeon Yellena Kim, Jaeyoung Kwon, Hak Cheol Kwon, C. Justin Lee, Dae Sik Jang

**Affiliations:** 1 Department of Biomedical and Pharmaceutical Sciences, Graduate School, Kyung Hee University, Seoul 02447, Republic of Korea; k_christina@khu.ac.kr (J.-Y.K.); allosori@khu.ac.kr (S.-R.S.); 2 Center for Cognition and Sociality, Institute for Basic Science, Daejeon 34126, Republic of Korea; ksy@ibs.re.kr (S.Y.K.); cjl@ibs.re.kr (C.J.L.); 3 KIST Gangneung Institute of Natural Products, Korea Institute of Science and Technology, Gangneung 25451, Republic of Korea; kjy1207@kist.re.kr (J.K.); hkwon@kist.re.kr (H.C.K.)

**Keywords:** *Aster koraiensis*, leaves, compositae, triterpenoidal saponins, SARS-CoV-2

## Abstract

Triterpenoidal saponins have been reported to be able to restrain SARS-CoV-2 infection. To isolate antiviral compounds against SARS-CoV-2 from the leaves of *Aster koraiensis*, we conducted multiple steps of column chromatography. We isolated six triperpenoidal saponins from *A*. *koraiensis* leaves, including three unreported saponins. Their chemical structures were determined using HR-MS and NMR data analyses. Subsequently, we tested the isolates to assess their ability to impede the entry of the SARS-CoV-2 pseudovirus (pSARS-CoV-2) into ACE2^+^ H1299 cells and found that five of the six isolates displayed antiviral activity with an IC_50_ value below 10 μM. Notably, one unreported saponin, astersaponin J (**1**), blocks pSARS-CoV-2 in ACE2^+^ and ACE2/TMPRSS2^+^ cells with similar IC_50_ values (2.92 and 2.96 μM, respectively), without any significant toxic effect. Furthermore, our cell-to-cell fusion and SARS-CoV-2 Spike-ACE2 binding assays revealed that astersaponin J inhibits membrane fusion, thereby blocking both entry pathways of SARS-CoV-2 while leaving the interaction between the SARS-CoV-2 Spike and ACE2 unaffected. Overall, this study expands the list of antiviral saponins by introducing previously undescribed triterpenoidal saponins isolated from the leaves of *A. koraiensis*, thereby corroborating the potency of triterpenoid saponins in impeding SARS-CoV-2 infection.

## 1. Introduction

Despite advances in vaccines and treatments, the persistent COVID-19 pandemic and the emergence of more contagious SARS-CoV-2 variants highlight the critical need for safe and effective antiviral solutions [1]. To date, research has increasingly concentrated on targeting membrane fusion, a crucial phase in SARS-CoV-2 infection, as a primary strategy to combat the virus [2,3,4]. SARS-CoV-2 begins its infection process by the spike glycoprotein (S protein) binding to the human angiotensin-converting enzyme 2 (ACE2) receptor, enabling entry into cells. This entry is aided either by the TMPRSS2 enzyme or through the mechanism of endocytosis [5,6]. After infiltrating inside the host cell, SARS-CoV-2 utilizes the cellular machinery to replicate, translating its RNA into essential nonstructural and structural proteins for viral assembly and proliferation [2]. These intricate processes, from entry to replication, are influenced by host cell proteases and the virus’s capacity to modify cellular functions [7].

Consequently, there has been a significant focus on discovering phytochemicals and herbal formulations that can inhibit these proteins’ activities and their interactions with cellular membranes, thereby preventing viral entry [8,9]. Studies reveal that phytochemicals can effectively target and disrupt the S-protein-mediated membrane fusion. For instance, curcumin, a diarylheptanoid from *Curcuma longa*, was found to impede SARS-CoV-2 entry in 293T/hACE/TMPRSS2 cells by blocking pseudovirus entry [10]. Similarly, emodin, an anthraquinone from *Rheum officinale*, significantly reduces the interaction between the S-protein and ACE2 [11] Moreover, our research, in conjunction with others, has identified a link between the chemical structure of oleanane-type saponins and their effectiveness as inhibitors of membrane fusion affected by SARS-CoV-2 [12,13].

*Aster koraiensis* Nakai (Synonym: *Gymnaster koraiensis*, Compositae), a native Korean plant primarily cultivated for ornamental use, has shown various pharmacological properties, such as anti-oxidant, anti-cancer, anti-inflammation, and hepatoprotective effects [14,15,16,17]. This plant also exhibits acyl CoA:cholesterol acyltransferase (ACAT) inhibition and affects the nuclear factor of activated T cell (NFAT) transcription factors [18,19]. A variety of secondary metabolites, including sesquiterpenes, triterpenes, flavonoids, caffeoylquinic acids, and benzofurans, have been isolated from *A*. *koraiensis* [15,16,17,18,19]. In our efforts to identify saponin-rich plants with antiviral capabilities against SARS-CoV-2, a 95% ethanol extract from *A*. *koraiensis* leaves was found particularly effective in obstructing viral entry. Notably, astersaponin I, a prominent triterpenoidal saponin in this extract, has been determined as the key inhibitor of SARS-CoV-2 [20].

Given the antiviral properties displayed by the *A. koraiensis* leaf extract, we speculated on its potential to contain a variety of saponins other than astersaponin I. Thus, this study aimed to isolate saponins previously unreported in *A. koraiensis* leaves and assess their antiviral potency. Repeated chromatographic processes successfully isolated three unreported triterpenoid saponins (**1**–**3**) and three known ones (**4**–**6**) from the 95% ethanol extract. Subsequently, we assessed their effect on the entry of SARS-CoV-2. Our study encompasses the saponin isolation procedure, elucidation of the structure of previously unreported compounds, and analysis of how these isolates affect SARS-CoV-2 infection.

## 2. Results and Discussion

### 2.1. Identification of Compounds 1–3 from the Leaves of A. koraiensis

**Figure 1:**
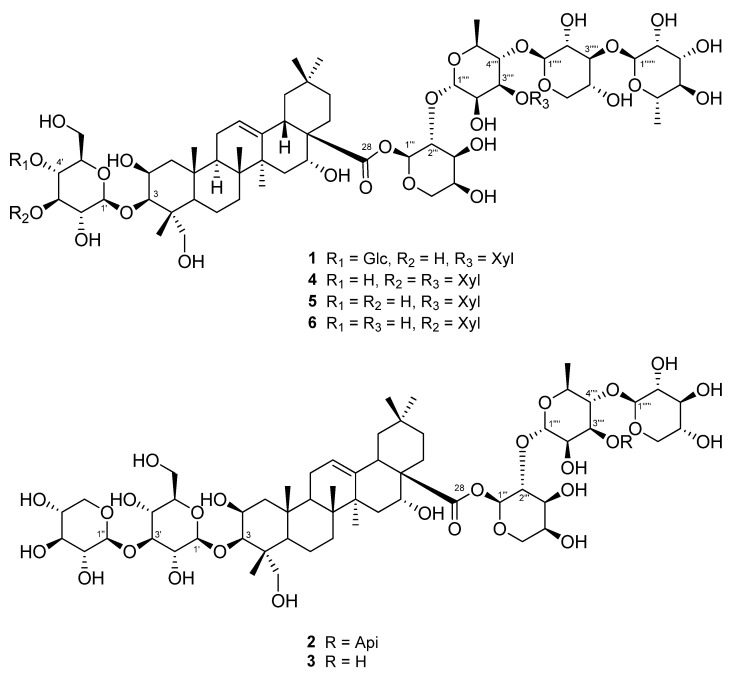
Structures of triterpenoid saponins **1**–**6** isolated from the leaves of *A. koraiensis*.

Three unreported (**1**–**3**) and three known triterpenoidal saponins (**4**–**6**) were isolated using a 95% EtOH extract of *A. koraiensis* leaves (Figure 1). Compounds **4**–**6** were identified as astersaponin I (**4**) [21], 3-*O*-*β*-d-glucopyranosyl-2*β*,3*β*,16*α*,23-tetrahydroxyolean-12-en-28-oic acid 28-*O*-*α*-l-rhamnopyranosyl-(1→3)-*β*-d-xylopyranosyl-(1→4)-[*β*-d-xylopyranosyl-(1→3)]-*α*-l-rhamnopyranosyl-(1→2)-*α*-l-arabinopyranoside (**5**) [22], and conyzasaponin J (**6**) [23] after comparing the spectroscopic data against those found in published data (Tables S1–S3). For the first time, we report the presence of compounds **5** and **6** in *A. koraiensis*.

**Figure 2:**
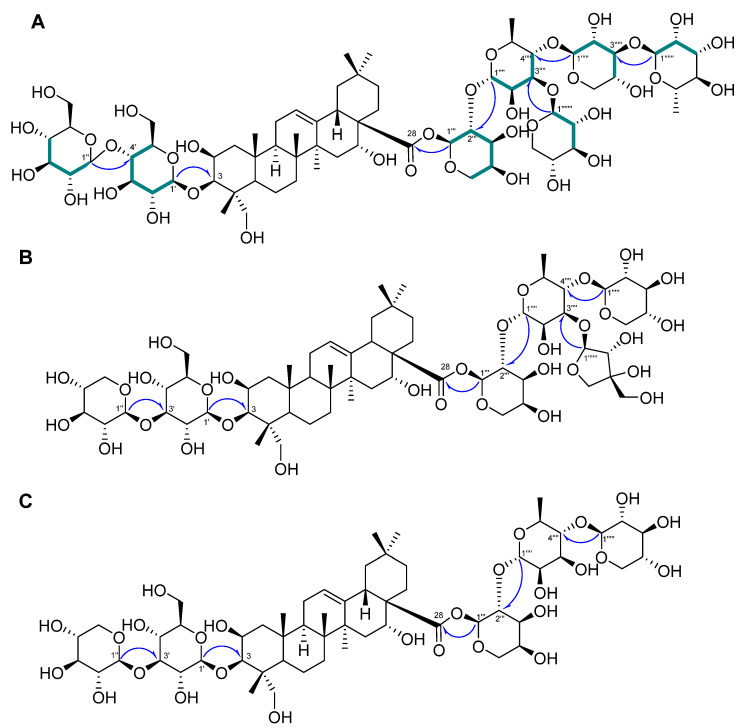
Key ^1^H−^13^C HMBC (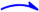) and HSQC-TOCSY (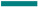) correlations of **1**–**3** (**A**–**C**).

Compound **1** was a white powder, and its chemical formula was deduced to be C_69_H_112_O_36_ by HR-ESI-MS (*m*/*z* 1517.7101 [M + H]^+^ calcd. 1517.7012) (Figure S1). The IR data showed absorbance bands at 3393, 2953, 1615, 1528, 1393, and 1040 cm^−1^, indicating that hydroxy and carbonyl groups are present in **1**. The ^1^H NMR spectrum of **1** showed eight methyl signals at *δ*_H_ 1.78 (3H, s, H-27), 1.74 (3H, d, *J* = 6.4 Hz, H-Rha-6‴′), 1.62 (3H, d, *J* = 6.4 Hz, H-Rha-6‴‴), 1.61 (3H, s, H-25), 1.35 (3H, s, H-24), 1.19 (3H, s, H-26), 1.15 (3H, s, H-30), and 0.99 (3H, s, H-29). Additionally, seven anomeric protons [*δ*_H_ 6.49 (1H, d, *J* = 1.8 Hz, H-Ara-1‴), 6.19 (1H, br s, H-Rha-1‴‴), 5.63 (1H, br s, H-Rha-1‴′), 5.39 (1H, d, *J* = 8.0 Hz, H-Xyl-1‴″), 5.14 (1H, d, *J* = 8.0 Hz, H-Glc-1′), 5.10 (1H, d, *J* = 8.0 Hz, H-Glc-1″), and 5.03 (1H, d, *J* = 7.5 Hz, H-Xyl-1‴‴′)] and one olefinic proton signal [5.62 (1H, br s, H-12)] were exhibited (Table 1; Figure S2). The sugars in **1** were suggested to be *β*-glucose, *β*-xylose, *α*-arabinose, and *α*-rhamnose via the interpretation of chemical shifts, coupling constants, and ^1^H-^1^H ROESY correlations at *δ*_H_ 6.49 (H-Ara-1‴) with 4.43 (H-Ara-5‴) and 4.60 (H-Ara-3‴), 6.19 (H-Rha-1‴‴) with 4.30 (H-Rha-3‴‴), 5.63 (H-Rha-1‴′) with 4.52 (H-Rha-4‴′), 5.33 (H-Xyl-1‴″) with 4.19 (H-Xyl-4‴″), 5.14 (H-Glc-1′) with 4.08 (H-Glc-3′) and 3.84 (H-Glc-5′), 5.10 (H-Glc-1″) with 4.27 (H-Glc-3″) and 4.04 (H-Glc-5″), and 5.03 (H-Xyl-1‴‴′) with 4.04 (H-Xyl-4‴‴′). The ^13^C, DEPT-135, and ^1^H-^13^C HSQC NMR spectra exhibited a total of 69 carbon signals with eight methyl (*δ*_C_ 33.5, 27.5, 25.0, 18.84, 18.80, 17.9, 17.6, and 15.3), 14 methylene (*δ*_C_ 67.0, 66.7, 64.8, 62.6, 62.2, 62.0, 46.8, 44.2, 35.9, 35.8, 33.1, 32.0, 23.8, and 17.8), seven anomeric carbon (*δ*_C_ 106.2, 105.4, 105.1, 104.8, 102.8, 101.1, and 93.6), an olefinic carbon (*δ*_C_ 123.9), and eight quaternary carbon (*δ*_C_ 176.3, 144.7, 49.4, 42.7, 42.0, 40.0, 36.8, and 30.8) signals, indicating that **1** is an oleanane-type triterpenoid glycoside with seven sugar moieties (Table 1; Figures S3 and S4). Through analysis of the ^1^H-^13^C HMBC spectrum of **1**, it was possible to identify the arrangement of sugars in **1**: *δ*_H_ 5.14 (H-Glc-1′)/*δ*_C_ 82.6 (C-3), *δ*_H_ 5.10 (H-Glc-1″)/*δ*_C_ 71.4 (C-4′), *δ*_H_ 6.49 (H-Ara-1‴)/*δ*_C_ 176.3 (C-28), *δ*_H_ 5.63 (H-Rha-1‴′)/*δ*_C_ 75.2 (C-2‴), *δ*_H_ 5.33 (H-Xyl-1‴″)/*δ*_C_ 71.5 (C-2‴′), *δ*_H_ 6.19 (H-Rha-1‴‴)/*δ*_C_ 75.9 (C-2‴″), and *δ*_H_ 5.03 (H-Xyl-1‴‴′)/*δ*_C_ 73.9 (C-3‴′) (Figure 2A and Figure S5). Furthermore, the chemical shifts of the carbons of each sugar in **1** could be assigned by an HSQC-TOCSY experiment (Figure 2A and Figure S7). Additionally, acid hydrolysis and a comparative experiment with authentic standards using LC-MS led to the identification of the sugar units as d-glucose, d-xylose, l-arabinose, and l-rhamnose. Comparative analysis with the reported spectroscopic data of tacacoside B_2_, previously isolated from *Sechium talamancense*, led to the determination of the chemical structure of **1** [20,24]. The distinguishing feature of **1** is the presence of an *β*-d-glucose unit at the C-4′ of the glucose at C-3, setting it apart from the previously identified tacacoside B_2_. Therefore, the chemical structure of **1** was elucidated as 3-*O*-*β*-d-glucopyranosyl-(1→4)-*β*-d-glucopyranosylpolygalacic acid-28-*O*-*α*-l-rhamnopyranosyl-(1→3)-*β*-d-xylopyranosyl-(1→4)-[*β*-d-xylopyranosyl-(1→3)]-*α*-l-rhamnopyranosyl-(1→2)-*α*-l-arabinopyranosyl-ester and named astersaponin J.

Compound **2** was a white powder, and its chemical formula was deduced to be C_62_H_100_O_31_ by HR-ESI-MS (*m*/*z* 1339.6171 [M − H]^−^ calcd. 1339.6176) (Figure S8). The ^1^H NMR spectrum of **2** exhibited a total of seven distinct methyl signals, which were observed at *δ*_H_ 1.78 (3H, s, H-27), 1.77 (3H, d, *J* = 6.0 Hz, H-Rha-6‴′), 1.61 (3H, s, H-25), 1.35 (3H, s, H-24), 1.17 (3H, s, H-26), 1.19 (3H, s, H-30), and 1.01 (3H, s, H-29) (Figure S7). Furthermore, six anomeric protons [*δ*_H_ 6.56 (1H, br s, H-Ara-1‴), 6.05 (1H, d, *J* = 5.0 Hz, H-Api-1‴‴), 5.64 (1H, br s, H-Rha-1‴′), 5.39 (1H, d, *J* = 8.0 Hz, H-Xyl-1‴″), 5.22 (1H, d, *J* = 7.5 Hz, H-Xyl-1″), and 5.17 (1H, d, *J* = 7.5 Hz, H-Glc-1′)] and an olefinic proton signal [5.64 (1H, br s, H-12)] were also observed in the ^1^H NMR spectrum (Table 2; Figure S9). Following analysis by the same method as for compound **1**, it was inferred that compound **2** contains one *β*-glucose, two *β*-xyloses, one *α*-arabinose, one *α*-rhamnose, and one *β*-apiose. The ROESY correlations were found at *δ*_H_ 6.56 (H-Ara-1‴) with 4.59 (H-Ara-3‴) and 4.44 (H-Ara-4‴), 5.64 (H-Rha-1‴′) with 4.46 (H-Rha-4‴′), 5.39 (H-Xyl-1‴″) with 4.26 (H-Rha-3‴′), 5.22 (H-Xyl-1″) with 4.16 (H-Xyl-3″), and 5.17 (H-Glc-1′) with 4.08 (H-Glc-3′) and 3.84 (H-Glc-5′). The ^13^C NMR spectrum of **2** displayed 62 carbon signals, with seven methyl signals (*δ*_C_ 33.6, 27.6, 25.2, 19.0, 18.1, 17.8, and 15.5), 15 methylene carbon signals (*δ*_C_ 74.9, 67.8, 67.6, 65.4, 64.7, 62.6, 62.5, 47.4, 44.8, 36.5, 36.4, 33.6, 32.5, 24.4, and 18.4), six methine anomeric carbons (*δ*_C_ 112.1, 106.7, 105.9, 105.5, 101.5, and 93.5), one olefinic carbon (*δ*_C_ 123.5), and nine quaternary carbons (*δ*_C_ 176.4, 144.8, 80.0, 50.0, 43.3, 42.6, 40.6, 37.4, and 31.3) (Table 2; Figure S10). Through the observed correlations at *δ*_H_ 5.17/*δ*_C_ 83.2 (C-3), *δ*_H_ 5.22/*δ*_C_ 88.0 (C-3′), *δ*_H_ 6.56/*δ*_C_ 176.4 (C-28), *δ*_H_ 5.64/*δ*_C_ 76.0 (C-2‴), *δ*_H_ 5.39/*δ*_C_ 72.0 (C-2‴′), and *δ*_H_ 6.05/*δ*_C_ 82.9 (C-3‴′) in the HMBC spectrum, the positions of the sugar moieties were determined (Figure 2B and Figure S12). The planar structure is similar to that of conyzasaponin P, except that oxygenated methine is located at the C16 position instead of methane [15]. The absolute configurations of the sugars in **2** were determined by acid hydrolysis and HPLC analysis as d-glucose, d-xylose, l-arabinose, l-rhamnose, and d-apiose. Thus, the structure of compound **2** was elucidated as 3-*O*-*β*-d-xylopyranosyl-(1→3)-*β*-d-glucopyranosylpolygalacic acid-28-*O*-*α*-l-rhamnopyranosyl-(1→3)-*β*-d-xylopyranosyl-(1→4)-[*β*-d-apiofuranosyl-(1→3)]-*α*-l-rhamnopyranosyl-(1→2)-*α*-l-arabinopyranosyl-ester and named astersaponin K.

The chemical composition of compound **3** was deduced to be C_57_H_92_O_27_ by HR-ESI-MS (*m*/*z* 1209.5897 [M + H]^+^ calcd. 1209.5899) (Figure S14). The ^1^H NMR spectrum of **3** showed five anomeric protons [*δ*_H_ 6.49 (1H, br s, H-Ara-1‴), 5.70 (1H, br s, H-Rha-1‴′), 5.20 (1H, d, *J* = 7.5 Hz, H-Xyl-5″), 5.14 (1H, d, *J* = 7.5 Hz, H-Glc-1′), and 5.09 (1H, d, *J* = 8.0 Hz, H-Xyl-1″)], and the ^13^C NMR spectrum of **3** exhibited a total of 57 carbon signals, suggesting that **3** has five sugar moieties (Table 3; Figures S15 and S16). The ^1^H and ^13^C NMR spectra of **3** were similar to those of **2** (Table 2 and Table 3). Careful analysis of the NMR spectroscopic data for **3** readily indicated that an apiose moiety was absent in **3** (Figure 1). The assignments of all of the functional groups and sugars in **3** were confirmed by 2D NMR experiments, and the absolute configuration of the sugar moiety was confirmed (using the same method as for compounds **1** and **2**) as d-glucose, d-xylose, l-arabinose, and l-rhamnose. Therefore, compound **3** was elucidated as 3-*O*-*β*-d-xylopranosyl-(1→3)-*β*-d-glucopyranosylpolygalacic acid-28-*O*-*β*-d-xylopyranosyl-(1→4)-*α*-l-rhamnopyranosyl-(1→2)-*α*-l-arabinopyranosyl-ester and named astersaponin L.

### 2.2. Astersaponin J Exhibits Comparable Inhibitory Activity against the Two SARS-CoV-2 Entry Pathways

**Figure 3:**
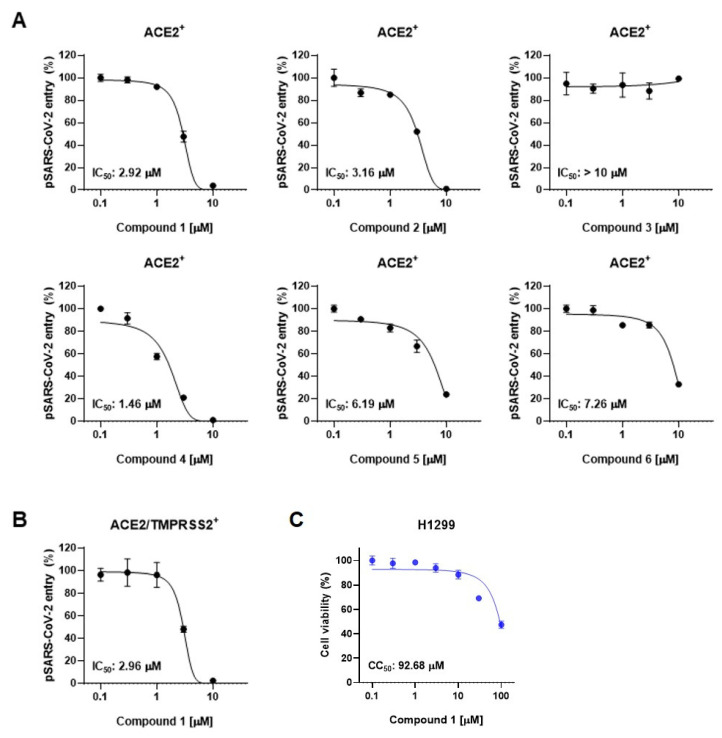
Triterpenoidal saponins from *A. koraiensis* effectively block SARS-CoV-2 infection: (**A**) The effects of compounds **1**–**6** from *A. koraiensis* on the entry of SARS-CoV-2 pseudovirus (pSARS-CoV-2) into ACE2^+^ H1299 cells. (**B**) pSARS-CoV-2 entry assay in ACE2/TMPRSS2^+^ H1299 cells. (**C**) Cell viability assays in H1299 cells for **1**. The results from the pSARS-CoV-2 entry assay are presented as representative data from triplicates. The error bars indicate the SEM (n > 3).

We next employed SARS-CoV-2 pseudotyped viruses (pSARS-CoV-2) to assess the inhibitory activity of compounds derived from the leaves of *A. koraiensis* against SARS-CoV-2. This biosafety-level-2 pseudovirus incorporates the S-protein onto HIV-based lentiviral particles, providing an effective tool for evaluating viral entry and screening antiviral compounds [25]. SARS-CoV-2 entry occurs through two distinct pathways: the endosomal pathway in ACE2-positive (ACE2^+^) H1299 cells and the TMPRSS2-mediated membrane fusion pathway in ACE2 and TMPRSS2 double-positive (ACE2/TMPRSS2^+^) H1299 cells. First, we investigated the effects of compounds **1**–**6** on pSARS-CoV-2 entry into ACE2-positive cells. The pSARS-CoV-2 entry assay revealed that all the compounds except for **3** exhibited dose-dependent inhibition of pSARS-CoV-2 entry into ACE2^+^ H1299 with an IC_50_ value of less than 10 μM (Figure 3A). Astersaponin I (**4**), reported as an inhibitor of infection with SARS-CoV-2 variants and syncytium formation in a previous report [13], showed the most potent inhibitory effect, with an observed IC_50_ value of 1.46 μM in this study. Astersaponins J (**1**) and K (**2**) also exhibited strong antiviral effects, with IC_50_ values of 2.92 and 3.16 μM. Astersaponin L (**3**) showed no antiviral activity until a concentration of 10 μM was reached (Figure 3A). Although less effective than astersaponin I (**4**), compounds **5** and **6** exhibit substantial inhibitory effects of 6.19 and 7.26 μM, respectively. Compounds **1**, **4**, and **5** are saponins with a pentasaccharide at C28, while compounds **2** and **6** have tetrasaccharide at C28, and compound **3** has a trisaccharide at C28.

Saponins, known for their soap-like ability to form micelles, are amphiphilic, surface-active compounds with a wide range of pharmacological properties [26]. These include hemolytic, insecticidal, anti-inflammatory, antitumor, antidiabetic, antifungal, anti-yeast, antibacterial, antiparasitic, antihyperlipidemic, and antioxidative effects [26]. Particularly, triterpenoid saponins and their derivatives are noted as potential antiviral agents due to their ability to block virus-host recognition across various virus species [27]. Since the antiviral effects of saikosaponins A, B_2_, C, and D have been reported as inhibitors of viral penetration into MRC-5 cells for human coronavirus (HCoV-229E), there has been significant research focusing on oleanane-type saponins and their role in blocking coronavirus membrane fusion [28]. Consistent with previous findings, our study indicates that oligosaccharides with four or more sugars are likely to bind to the C28 of triterpenoidal saponins, thereby obstructing the entry of pSARS-CoV-2 into host cells [12,27,29,30]. Next, we chose astersaponin J (**1**) to conduct a more detailed investigation, because this compound is a newly reported saponin and the most potent (except for astersaponin I, **4**) in this study. The results of the pSARS-CoV-2 entry assay further demonstrated that astersaponin J (**1**) exhibits comparable efficiency in inhibiting pSARS-CoV-2 entry into ACE2/TMPRSS2^+^ H1299 and ACE2^+^ H1299 cells, with an IC_50_ value of 2.96 μM (Figure 3B). Importantly, treatment with astersaponin J (**1**) up to a concentration of 10 μM did not result in cytotoxicity in H1299 cells (Figure 3C).

It is noteworthy that our previous study, employing authentic SARS-CoV-2 and variants, demonstrated that astersaponin I (**4**) displays an inhibitory activity 3.9 to 8.6 times stronger than that of chloroquine, a reported SARS-CoV-2 entry inhibitor [12]. Since astersaponin J (**1**) has similar anti-SARS-CoV-2 properties to astersaponin I (**4**), astersaponin J (**1**) might surpass chloroquine in efficacy.

### 2.3. Astersaponin J Effectively Inhibits SARS-CoV-2 Entry by Blocking S-Protein-Mediated Viral Membrane Fusion

**Figure 4:**
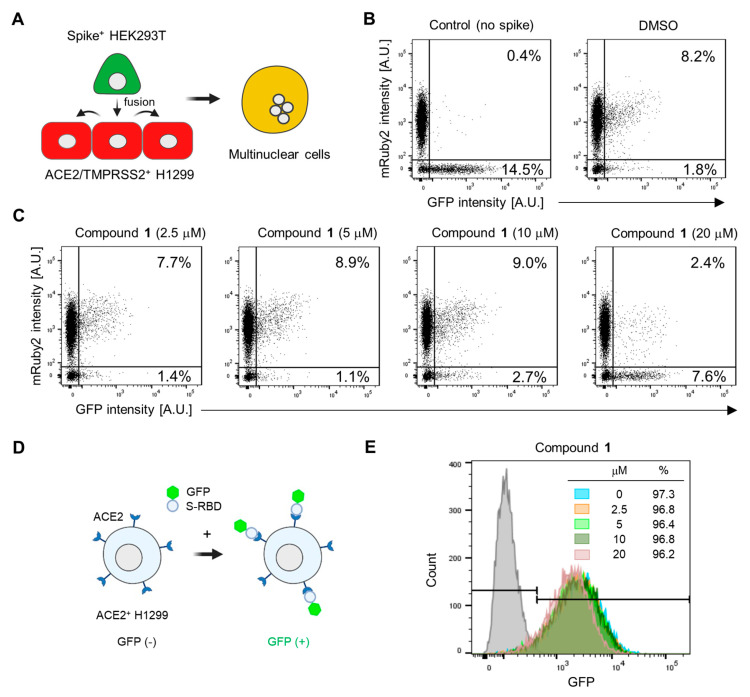
Astersaponin J (**1**) hinders SARS-CoV-2 envelope fusion with the host cell membrane without affecting the S-ACE2 interaction: (**A**) Schematic illustration depicting the process of cell fusion mediated by the SARS-CoV-2 S protein. In this process, a cell suspension of Spike+ HEK293T cells, which stably express GFP, is added to a monolayer of ACE2/TMPRSS2^+^ H1299 cells, which exhibit mRuby fluorescence. This interaction between the two cell types results in cell-to-cell fusion, generating multinuclear cells. (**B**) The number of cells that were double-positive for GFP and mRuby, indicating cell-to-cell fusion, was quantified using flow cytometry. A control experiment was conducted using HEK293T cells expressing only GFP (without the spike protein). (**C**) ACE2/TMPRSS2^+^ H1299 cells were pre-treated with the indicated concentrations of **1** for 1 h prior to adding Spike+ HEK293T cells, followed by flow cytometry. The result are presented as representative data from triplicates. (**D**) A diagram depicting the interaction between the SARS-CoV-2 spike receptor binding domain (RBD) fused to the GFP (S-RBD-GFP) and ACE2 protein, which is overexpressed in H1299 cells. (**E**) The effect of **1** on the interaction between S-RBD-GFP and ACE2 on the surface of H1299 cells, observed by flow cytometry after treatment with the predetermined concentrations of **1** for 1 h. The grey peaks in the flow cytometry graphs represent control experiments conducted without the addition of RBD-GFP.

Based on our observation that astersaponin J (**1**) significantly inhibits both primary SARS-CoV-2 entry routes, we hypothesized that astersaponin J (**1**) may target the shared entry process, such as the membrane fusion between viral and host membranes. To study S-mediated membrane fusion as a representative model of viral entry, we used two stable cell lines: Spike-HEK293T cells, which overexpress S-protein with EGFP from a single bicistronic mRNA in HEK293T cells, and ACE2/TMPRSS2^+^ H1299 cells, which overexpress mRuby [13]. Introducing Spike-HEK293 cells into a monolayer of ACE2/TMPRSS2^+^ H1299 cells swiftly triggers cell-to-cell fusion. The presence of S-proteins on the surface of the fused hybrid cells facilitates continuous merging with adjacent ACE2/TMPRSS2^+^ H1299 cells, resulting in the formation of multinucleated cells (Figure 4A). Flow cytometry analysis was conducted to assess the extent of cell-to-cell fusion after co-culturing these two cell types in a 1:10 ratio. This analysis indicated that about 8% of the total cells were double-positive for mRuby and GFP, implying that 80% of the Spike-HEK293 cells participated in the fusion process (Figure 4B).

In contrast, when control GFP-HEK293 cells (lacking S-protein) were co-cultured with ACE2/TMPRSS2^+^ cells, double-positive cells were barely detected, highlighting the requirement of S-protein for cell-to-cell fusion (Figure 4B). To assess the potential anti-fusion activity of astersaponin J (**1**), we pre-treated the cells with varying concentrations of astersaponin J (**1**) for 1 h before the addition of Spike-HEK293 cells. Remarkably, at a concentration of 20 μM, astersaponin J (**1**) demonstrated a robust blockade of the fusion event. Only 24% of the cells participated in the generation of cell fusion hybrids, whereas 76% of the cells did not engage in fusion (Figure 4C). Due to the significant size difference between HEK293T cells and viral particles, the fusion efficiency between HEK293T cells and H1299 cells was much higher than between viral particles and H1299 cells. As a result, a higher concentration of astersaponin J (**1**) was needed to block this fusion process compared to that required to inhibit viral fusion (Figure 3A and Figure 4C). Next, we evaluated the effect of astersaponin J (**1**) on the interaction between the SARS-CoV-2 S protein and ACE2. To assess this, we used a recombinant protein comprising the receptor-binding domain (RBD) of the S-protein fused with GFP (S-RBD-GFP) and introduced it into ACE2^+^ H1299 cells (Figure 4D). Flow cytometry analysis revealed that more than 97% of the cells exhibited binding of S-RBD-GFP (Figure 4E), indicating the attachment of the SARS-CoV-2 S protein to the cellular receptor required for viral entry. Notably, when ACE2^+^ H1299 cells were pre-treated with astersaponin J (**1**), there was no notable impact on the binding of S-RBD-GFP to ACE2 within the applied concentration range, although a minor peak shift was observed at 20 μM in the binding profile. These findings indicate that astersaponin J (**1**) specifically disrupts the S-protein-mediated membrane fusion process without altering the protein–protein interaction between the S-protein and ACE2. This distinct mechanism of action contributes to effectively inhibiting both major entry pathways of SARS-CoV-2.

## 3. Materials and Methods

### 3.1. General Experimental Procedures

The general experimental procedures are provided in Supplementary Data.

### 3.2. Plant Material

The *A. koraiensis* leaves were collected in Pyeongchang, Republic of Korea, in 2017. The plant’s origin was verified by Prof. Dae Sik Jang (D.S.J.), and a reference sample (ASKO1-2017) has been stored at the College of Pharmacy, Kyung Hee University, Republic of Korea.

### 3.3. Extraction and Isolation

The dried leaves of *A. koraiensis* (5.0 kg) were subjected to extraction twice with 25 L of 95% EtOH (70 °C, 3 h). The extraction mixture was concentrated by a rotary evaporator. The 95% EtOH extract (500.0 g) was loaded with Diaion HP-20 CC and eluted with a gradient solvent system (acetone:H_2_O = 0:1–1:0, *v*/*v*; 9.8 × 63.0 cm) to generate 28 subfractions (Fr. C1–Fr. C28). Fr. C11 (10.0 g) was separated into seven subfractions (Fr. C11-1–Fr. C11-7) using a Sephadex LH-20 CC (acetone:H_2_O = 1:1, *v*/*v*; 3.6 × 65.0 cm). Fr. C11-1 (1.06 g) was further fractionated by silica gel CC to produce three subfractions (Fr. C11-1-1–Fr. C11-1-3, methylene chloride:methanol:H_2_O = 10:0:0–0:9:1, *v*/*v*). Compounds **2** (40.6 mg, 95%) and **5** (10.8 mg, 95%) were obtained from Fr. C11-1-2 by repeated Sephadex LH-20 CC with an isocratic solvent system (acetone:H_2_O = 1:1, *v*/*v*; 3.6 × 65.0 cm). Furthermore, compounds **1** (32.0 mg, *t*_R_ = 19.5 min, 98%), **3** (6.8 mg, *t*_R_ = 3.8 min, 97%), **4** (99.6 mg, *t*_R_ = 21.1 min, 97%), and **6** (15.1 mg, *t*_R_ = 22.0 min, 99%) were purified using preparative HPLC with a Gemini 5 µm NX-C18 110A column (acetonitrile:H_2_O = 20:60–40:60, *v*/*v*) from Fr. C11-1-1 (Scheme S1). We detected the compounds with a photodiode-array detector at 210 nm.

Astersaponin J (**1**): white powder; HR-ESI-MS (positive mode) *m*/*z* = 1517.7101 [M + H]^+^ (calcd. for C_69_H_113_O_36_, 1517.7012); m.p. 196 °C; ${\left[\alpha \right]}_{D}^{25}$: −51.3 (c 0.1, MeOH); IR (ATR) ν_max_ 3393, 2953, 1615, 1528, 1393, 1040 cm^−1^; ^1^H and ^13^C NMR data, see Table 1.

Astersaponin K (**2**): pale yellowish powder; HR-ESI-MS (negative mode) *m*/*z* = 1339.6171 [M − H]^−^ (calcd. for C_62_H_99_O_31_, 1339.6176); m.p. 162 °C; ${\left[\alpha \right]}_{D}^{25}$: −99.3 (*c* 0.1, MeOH); IR (ATR) ν_max_ 3382, 2918, 1639, 1408, 1043 cm^−1^; ^1^H and ^13^C NMR data, see Table 2.

Astersaponin L (**3**): pale yellowish powder; HR-ESI-MS (positive mode) *m*/*z* = 1209.5897 [M + H]^+^ (calcd. for C_57_H_93_O_27_, 1209.5899); m.p. 186 °C; ${\left[\alpha \right]}_{D}^{25}$: −42.6 (*c* 0.1, MeOH); IR (ATR) ν_max_ 3395, 2928, 1559, 1407, 1030 cm^−1^; ^1^H and ^13^C NMR data, see Table 3.

### 3.4. Absolute Configurations of Sugars

To perform the absolute structural analysis of sugars, acid hydrolysis was conducted. Each sample (**1**–**3**; 3.0 mg) was diluted in 2 M D_2_SO_4_ and heated at 60 °C for 3 h. Subsequently, the reaction crude was concentrated and partitioned with H_2_O and BuOH. The water-soluble fraction was dissolved in a solution containing 10 mg/mL l-cysteine methyl ester and pyridine, and the reaction was conducted at 60 °C in a water bath for 1 h. Then, 20.0 µL of *o*-tolyl isothiocyanate was added for the consequent reaction at 60 °C for 1 h. The identification of sugars was performed using LC-ESI-MS.

A Thermo Hypersil GOLD C18 column (Thermo Fisher Scientific, Waltham, MA, USA) was employed to separate the derivatized mixture at 35 °C for 20 min under isocratic conditions with a mobile phase consisting of 0.5% formic acid (FA) in H_2_O and 0.5% FA in acetonitril in a ratio of 80:20. The determination of l-arabinose (*t*_R_ 9.0 min), d-glucose (*t*_R_ 11.0 min), d-xylose (*t*_R_ 12.2 min), l-rhamnose (*t*_R_ 18.3 min), and d-apiose (*t*_R_ 16.8 min) was achieved by comparing their retention times (*t*_R_) and full MS spectrum with authentic standards (Figures S20–S23).

### 3.5. Cell Culture

H1299 cells (KCLB, Seoul, Republic of Korea, 25803) were acquired from the Korean Cell Line Bank and maintained in RPMI 1640 medium (Gibco, Grand Island, NY, USA). HEK293T cells (CRL-3216) were purchased from the American Type Culture Collection and cultured in Dulbecco’s Modified Eagle’s Medium (DMEM; Corning, Glendale, AZ, USA). Both cell lines were maintained with 10% fetal bovine serum (FBS) (Gibco, Grand Island, NY, USA) and 1× penicillin–streptomycin solution (HyClone, Logan, UT, USA). The cells were cultured at 37 °C in a humidified incubator with 5% CO_2_.

### 3.6. Cell Viability Assay

H1299 cells were plated in a 96-well plate at a density of 5 × 10^3^ cells per well. The following day, the cells were treated with various concentrations of astersaponin J as indicated. After 24 h of incubation, WST-8 solution (Biomax, Guri-si, Gyeonggi-do, Republic of Korea) was added to each well and incubated for 2 h at 37 °C in a CO_2_ incubator. The absorbance of each well was measured at 450 nm using a SpectraMax iD5 Multi-Mode Microplate Reader (Molecular Devices, San Jose, CA, USA). To determine the 50% cytotoxic concentration (CC_50_), the mean absorbance values of the drug-treated samples were compared to those of cells treated with DMSO, and the ratio between the two was calculated.

### 3.7. Generation of Stable Cell Lines

H1299 cells stably expressing ACE2 (ACE2^+^) or ACE2 plus TMPRSS2 (ACE2/TMPRSS2^+^), ACE2/TMPRSS2^+^ H1299 cells expressing mRuby2, and HEK293T cells co-expressing S-protein and GFP (Spike^+^ HEK293T) were generated by lentiviral transduction as previously described [12].

### 3.8. SARS-CoV-2 S-Pseudotyped Lentivirus Production and pSARS-CoV-2 Entry Assay

To generate SARS-CoV-2 S-pseudotyped lentiviruses (pSARS-CoV-2), we employed a second-generation lentiviral packing system following a previously described method [13]. Briefly, HEK293T cells were transfected with a lentiviral plasmid containing the fly luciferase gene, psPAX2 packing plasmid, and SARS-CoV-2 S plasmid using Lipofectamine 3000 transfection reagent (Invitrogen, Carlsbad, CA, USA) according to the manufacturer’s instructions. Culture supernatants were collected at 24 h and 48 h post-transfection, centrifuged to remove cellular debris, and stored at 4 °C until further use. For the pSARS-CoV-2 entry assay, supernatants containing pSARS-CoV-2 virus particles were added to ACE2^+^ and ACE2/TMPRSS2^+^ H1299 cells, along with each compound. Following 24 h of incubation, the efficiency of viral entry was measured by quantifying the firefly luciferase activity in the cell lysates using a luciferase assay system (Promega, Madison, WI, USA) and SpectraMax iD5 Multi-Mode Microplate Reader (Molecular Devices, San Jose, CA, USA). The dose–response curves were created utilizing Prism v.9.0.0 software (GraphPad, CA, USA). The IC_50_ and CC_50_ values were determined through nonlinear regression analysis, employing the log of inhibitor concentration versus response using a variable slope equation: Y = bottom + (top − bottom)/(1 + 10[(logIC50 − X)^HillSlope^]). All IC_50_ and CC_50_ values were obtained in triplicate measurements.

### 3.9. Cell-to-Cell Fusion Assay

ACE2/TMPRSS2^+^ H1299 cells co-expressing mRuby2 were seeded in a 12-well plate at a density of 2 × 10^5^ cells per well and allowed to adhere overnight. The following day, the cell cultures were treated with either DMSO or the indicated concentration of astersaponin J for 1 h. Subsequently, Spike^+^ HEK293T cells co-expressing GFP were added to the wells at a density of 2 × 10^4^ cells. After incubation for 1 h, the co-cultures were harvested using trypsin-EDTA (Gibco, USA). Flow cytometry analysis was performed using an LSRFortessa™ flow cytometer (BD Biosciences, San Jose, CA, USA) to determine the percentage of cells that were double-positive for GFP and mRuby, indicating cell-to-cell fusion. A total of 1 × 10^4^ cells were analyzed, and the data were analyzed using FlowJo (v9) software (Tree Star Inc, Ashland, OR, USA).

### 3.10. SARS-CoV-2 S and ACE2 Binding Assay

S-RBD-GFP, a fusion protein combining the receptor binding domain (RBD) of the SARS-CoV-2 Spike protein and GFP, was generated using Expi293F cells, following a previously described method [12]. ACE2^+^ H1299 cells were treated with different concentrations of astersaponin J for 1 h. Subsequently, a medium containing S-RBD-GFP was added to the cells at a ratio of 10:1 and incubated for an additional 10 min at 37 °C in a CO_2_ incubator. The mixture of ACE2^+^ H1299 cells and S-RBD-GFP was then washed with PBS containing 1% bovine serum albumin, and flow cytometry analysis was performed using an LSRFortessa flow cytometer (BD Biosciences). Approximately 10,000 cells were analyzed to determine the binding of S-RBD-GFP to ACE2 on the surface of H1299 cells. The obtained data were analyzed using FlowJo software (BD Life Sciences).

## 4. Conclusions

To identify novel natural antiviral substances against SARS-CoV-2, we isolated and characterized three previously undescribed triterpenoidal saponins (**1**–**3**) and three known saponins (**4**–**6**) from the leaves of *A. koraiensis*. All triterpenoidal saponins with penta or tetraoligosaccharides at C28 (**1**, **2**, and **4**–**6**) inhibited the entry of pSARS-CoV-2 into ACE2^+^ H1299 cells with an IC_50_ value below 10 μM. Specifically, astersaponin J (**1**) demonstrated notable effectiveness in impeding pSARS-CoV-2 entry into ACE2/TMPRSS2^+^ H1299 cells, with an IC_50_ value of 2.96 μM. Moreover, we identified the mode of action of astersaponin J (**1**), which involves inhibiting S protein-mediated viral membrane fusion. The effectiveness of triterpenoidal saponins in inhibiting the entry of pSARS-CoV-2 into host cells seems to depend on the number of sugars present at the C28 location, with a minimum of four sugars expected to lead to desirable outcomes. In conclusion, the triterpenoidal saponins found in *A. koraiensis* leaves, including the astersaponin J (**1**) newly identified in this study, along with the previously discovered astersaponin I (**4**), exhibit significant potential as antiviral agents against SARS-CoV-2, thereby providing promising possibilities for dealing with the COVID-19 pandemic and potential viral outbreaks in the future.

## Figures and Tables

**Table 1:** ^1^H and ^13^C NMR spectroscopic data of compound **1** (*δ* in ppm, pyridine-*d*_5_, 800 and 200 MHz).

Position	Aglycon		Position	Sugar	
	*δ*_H_ Multi (*J* in Hz)	*δ*_C_		*δ*_H_ Multi (*J* in Hz)	*δ*_C_
1	1.26/2.30 ^a^	44.7	Glc-1′	5.14 d (8.0)	105.4
2	4.77 ^a^	71.1	Glc-2′	4.05 ^a^	74.2
3	4.32 ^a^	83.0	Glc-3′	4.08 ^a^	78.5
4	—	43.1	Glc-4′	4.12 ^a^	71.4
5	1.71 m	47.9	Glc-5′	3.84 ^a^	77.7
6	1.78 m/1.83 m	18.2	Glc-6′	4.28/4.42 ^a^	62.0
7	1.55 m/1.74 m	33.5	Glc-1″	5.10 d (8.0)	106.1
8	—	40.4	Glc-2″	4.07 ^a^	75.6
9	1.80 m	47.8	Glc-3″	4.27 ^a^	78.3
10	—	37.2	Glc-4″	4.21 ^a^	71.6
11	1.80 m/2.05 m	24.2	Glc-5″	4.04 ^a^	78.8
12	5.62 br s	124.3	Glc-6″	4.32/4.55 ^a^	62.6
13	—	144.6	Ara-1‴	6.49 d (1.8)	93.5
14	—	42.4	Ara-2‴	4.53 ^a^	75.2
15	1.74 m/2.22-2.43 ^a^	36.3	Ara-3‴	4.60 ^a^	69.4
16	5.26 br s	74.3	Ara-4‴	4.43 ^a^	65.3
17	—	49.7	Ara-5‴	3.92/4.47 ^a^	62.2
18	3.60 m	41.4	Rha-1‴′	5.63 br s	101.1
19	1.34/2.76 ^a^	47.2	Rha-2‴′	4.77 ^a^	71.5
20	—	31.2	Rha-3‴′	4.54 dd (9.6, 3.2)	73.9
21	1.29/2.22–2.43 ^a^	36.2	Rha-4‴′	4.52 ^a^	83.6
22	2.14/2.26 ^a^	32.4	Rha-5‴′	4.40 ^a^	68.6
23	4.30 br d (10.4)/3.62 br d (10.4)	65.2	Rha-6‴′	1.74 d (6.4)	18.80
24	1.35 s	15.3	Xyl-1‴″	5.39 d (8.0)	104.9
25	1.61 s	17.6	Xyl-2‴″	3.92 ^a^	75.9
26	1.19 s	17.9	Xyl-3‴″	4.19 ^a^	82.2
27	1.78 s	27.5	Xyl-4‴″	4.08 ^a^	68.8
28	—	176.3	Xyl-5‴″	3.46 dd (12.0, 0.8)/4.04 ^a^	67.0
29	0.99 s	33.5	Rha-1‴‴	6.19 br s	102.6
30	1.15 s	25.0	Rha-2‴‴	4.78 dd (3.6, 1.8)	72.3
			Rha-3‴‴	4.58 dd (8.8, 3.2)	72.4
			Rha-4‴‴	4.30 m^a^	72.8
			Rha-5‴‴	4.95 dq (9.6, 6.4)	69.1
			Rha-6‴‴	1.64 d (6.4)	18.84
			Xyl-1‴‴′	5.03 d (8.0)	106.2
			Xyl-2‴‴′	3.94 ^a^	74.2
			Xyl-3‴‴′	3.96 ^a^	77.8
			Xyl-4‴‴′	4.04 ^a^	69.6
			Xyl-5‴‴′	3.56 dd (14.4, 4.8)/4.04 ^a^	66.7

^a^ Overlapped signals.

**Table 2:** ^1^H and ^13^C NMR spectroscopic data of compound **2** (*δ* in ppm, pyridine-*d*_5_, 500 and 125 MHz).

Position	Aglycon		Position	Sugar	
	*δ*_H_ Multi (*J* in Hz)	*δ*_C_		*δ*_H_ Multi (*J* in Hz)	*δ*_C_
1	1.26/2.30 ^a^	44.8	Glc-1′	5.17 d (7.5)	105.9
2	4.80 m	71.3	Glc-2′	4.04 ^a^	74.7
3	4.35 m	83.2	Glc-3′	4.08 ^a^	88.0
4	—	43.3	Glc-4′	4.13 ^a^	69.7
5	1.71 ^a^	48.1	Glc-5′	3.84 ddd (9.0, 4.5, 2.0)	78.3
6	1.78/1.83 ^a^	18.4	Glc-6′	4.28/4.41 ^a^	62.6
7	1.55/1.74 ^a^	33.6	Xyl-1″	5.22 d (7.5)	106.7
8	—	40.6	Xyl-2″	4.02 ^a^	75.7
9	1.80 ^a^	48.0	Xyl-3″	4.16 ^a^	78.6
10	—	37.4	Xyl-4″	4.18 ^a^	71.2
11	1.80/2.05 ^a^	24.4	Xyl-5″	3.70 m/4.32 (11.5, 4.0)	67.8
12	5.64 br s	123.5	Ara-1‴	6.56 br s	93.5
13	—	144.8	Ara-2‴	4.48 ^a^	76.0
14	—	42.6	Ara-3‴	4.59 ^a^	69.1
15	1.74/2.22–2.43^a^	36.5	Ara-4‴	4.44 ^a^	65.7
16	5.25 m	74.5	Ara-5‴	3.97/4.63 ^a^	62.5
17	—	50.0	Rha-1‴′	5.64 br s	101.5
18	3.61 ^a^	41.6	Rha-2‴′	4.73 ^a^	72.0
19	1.34/2.76 ^a^	47.4	Rha-3‴′	4.36 ^a^	82.9
20	—	31.3	Rha-4‴′	4.46 ^a^	78.6
21	1.29/2.22–2.43 ^a^	36.4	Rha-5‴′	4.32 ^a^	69.3
22	2.14/2.26	32.5	Rha-6‴′	1.77 d (6.0)	19.0
23	3.68 d (9.5)/4.31 d (9.5)	65.4	Xyl-1‴″	5.39 d (8.0)	105.5
24	1.35 s	15.5	Xyl-2‴″	3.94 ^a^	75.9
25	1.61 s	17.8	Xyl-3‴″	4.26 ^a^	78.3
26	1.17 s	18.1	Xyl-4‴″	4.08 ^a^	71.6
27	1.78 s	27.6	Xyl-5‴″	3.39 t (11.0)/4.14 ^a^	67.6
28	—	176.4	Api-1‴‴	6.05 d (5.0)	112.1
29	1.01 s	33.6	Api-2‴‴	4.78 br d (5.0)	77.8
30	1.19 s	25.2	Api-3‴‴	—	80.0
			Api-4‴‴	4.18 br d (9.5)/4.56 br d (9.5)	74.9
			Api-5‴‴	4.06 br d (10.5)	64.7

^a^ Overlapped signals.

**Table 3:** ^1^H and ^13^C NMR spectroscopic data of compound **3** (*δ* in ppm, pyridine-*d*_5_, 500 and 125 MHz).

Position	Aglycon		Position	Sugar	
	*δ*_H_ Multi (*J* in Hz)	*δ*_C_		*δ*_H_ Multi (*J* in Hz)	*δ*_C_
1	1.26/2.29 ^a^	44.2	Glc-1′	5.14 d (7.5)	105.4
2	4.82 ^a^	70.7	Glc-2′	4.03 m	74.4
3	4.32 ^a^	82.9	Glc-3′	4.07 t (9.0)	87.6
4	—	42.8	Glc-4′	4.13 br t (9.0)	69.4
5	1.70 m	47.6	Glc-5′	3.84 ddd (9.0, 4.5, 2.0)	77.9
6	1.78/1.82 ^a^	18.0	Glc-6′	4.28 dd (11.5, 4.5)/4.40 m	62.2
7	1.54 m/1.73 ^a^	33.2	Xyl-1″	5.20 d (7.5)	106.3
8	—	40.1	Xyl-2″	4.00 t (8.0)	75.3
9	1.80 ^a^	47.7	Xyl-3″	4.15 ^a^	78.2
10	—	36.9	Xyl-4″	4.16 ^a^	70.9
11	1.80 ^a^/2.05 m	24.0	Xyl-5″	3.70 t (11.0)/4.30 ^a^	67.4
12	5.62 br s	123.0	Ara-1‴	6.49 br s	93.4
13	—	144.3	Ara-2‴	4.52 ^a^	75.2
14	—	42.2	Ara-3‴	4.53 ^a^	69.9
15	1.74 m/2.23–2.45 ^a^	36.1	Ara-4‴	4.39 ^a^	66.1
16	5.28 m	73.9	Ara-5‴	3.95 dd (11.0, 4.0)/4.55 ^a^	63.0
17	—	49.5	Rha-1‴′	5.70 br s	101.0
18	3.38 dd (5.0, 14.0)	41.2	Rha-2‴′	4.82 br dd (3.0, 1.5)	71.9
19	1.34 ^a^/2.75 m	47.0	Rha-3‴′	4.50 ^a^	72.7
20	—	30.8	Rha-4‴′	4.33 ^a^	83.9
21	1.29 m/2.23–2.45 ^a^	35.9	Rha-5‴′	4.35 ^a^	68.6
22	2.18 m/2.27 ^a^	32.0	Rha-6‴′	1.72 d (5.5)	18.4
23	3.65 d (10.0)/4.35 d (10.0)	65.1	Xyl-1‴″	5.09 d	106.8
24	1.34 s	15.0	Xyl-2‴″	4.00 t (8.5)	76.2
25	1.60 s	17.3	Xyl-3‴″	4.19 ^a^	83.3
26	1.18 s	17.6	Xyl-4‴″	4.10 ^a^	69.3
27	1.77 s	27.1	Xyl-5‴″	3.44 t (11.0)/4.18 ^a^	67.3
28	—	175.9			
29	0.99 s	33.2			
30	1.15 s	24.7			

^a^ Overlapped signals.

## Data Availability

Data are contained within the article and Supplementary Materials.

## Supplementary Materials

The following supporting information can be downloaded at https://www.mdpi.com/article/10.3390/plants13020303/s1: Figure S1. HR-ESI-MS full-spectrum (A) and zoom-spectrum (B) of compound **1**; Figure S2. ^1^H NMR spectrum of compound **1** (800 MHz, pyridine-*d*_5_); Figure S3. ^13^C NMR spectrum of compound **1** (200 MHz, pyridine-*d*_5_); Figure S4. ^1^H-^13^C HSQC spectrum of compound **1**; Figure S5. ^1^H-^13^C HMBC spectrum of compound **1**; Figure S6. ^1^H-^1^H ROESY spectrum of compound **1**; Figure S7. HSQC-TOSCY spectrum of compound **1**; Figure S8. HR-ESI-MS spectrum of compound **2**; Figure S9. ^1^H NMR spectrum of compound **2** (500 MHz, pyridine-*d*_5_); Figure S10. ^13^C NMR spectrum of compound **2** (125 MHz, pyridine-*d*_5_); Figure S11. ^1^H-^13^C HSQC spectrum of compound **2**; Figure S12. ^1^H-^13^C HMBC spectrum of compound **2**; Figure S13. ^1^H-^1^H ROESY spectrum of compound **2**; Figure S14. HR-ESI-MS spectrum of compound **3**; Figure S15. ^1^H NMR spectrum of compound **3** (500 MHz, pyridine-*d*_5_); Figure S16. ^13^C NMR spectrum of compound **3** (125 MHz, pyridine-*d*_5_); Figure S17. ^1^H-^13^C HSQC spectrum of compound **3**; Figure S18. ^1^H-^13^C HMBC spectrum of compound **3**; Figure S19. ^1^H-^1^H NOESY spectrum of compound **3**; Figure S20. A: extracted ion chromatograms (EICs) of derivatized glucose and hydrolyzed samples in negative mode (mass range of *m*/*z* 444.50–445.50, d-glucose: *t*_R_ 11.0 min), B: full MS spectrum of derivatized glucose and hydrolyzed samples; Figure S21. A: extracted ion chromatograms (EICs) of derivatized rhamnose and hydrolyzed samples in negative mode (mass range of *m*/*z* 428.50–429.50, l-rhamnose: *t*_R_ 18.31 min), B: full MS spectrum of derivatized rhamnose and hydrolyzed samples; Figure S22. extracted ion chromatograms (EICs) of derivatized pentose and hydrolyzed samples in negative mode (mass range of *m*/*z* 414.50–415.50, d-apiose: *t*_R_ 16.82 min; l-arabinose: *t*_R_ 9.01 min; l-xylose: *t*_R_ 12.22 min); Figure S23. Full MS spectrum of derivatized apiose and hydrolyzed samples; Figure S24. Full MS spectrum of derivatized arabinose and hydrolyzed samples; Figure S25. Full MS spectrum of derivatized xylose and hydrolyzed samples; Scheme S1. Isolation scheme of compounds **1**–**6** from the 95% EtOH extract of *A*. *koraiensis* leaves; Table S1. ^1^H and ^13^C NMR spectroscopic data of compound **4** (*δ* in ppm, pyridine-*d*_5_, 500 and 125 MHz); Table S2. ^1^H and ^13^C NMR spectroscopic data of compound **5** (*δ* in ppm, pyridine-*d*_5_, 500 and 125 MHz); Table S3. ^1^H and ^13^C NMR spectroscopic data of compound **6** (*δ* in ppm, pyridine-*d*_5_, 500 and 125 MHz).
