# Supplementary material for: Triterpenoidal Saponins from the Leaves of Aster koraiensis Offer Inhibitory Activities against SARS-CoV-2
Source: Plants (Basel). 2024 Jan 19;13(2):303. doi: 10.3390/plants13020303 (PMC10819127; doi:10.3390/plants13020303)
Supplement: Supplementary file 1 [file plants-13-00303-s001.zip › plants-2783287-supplementary.pdf]

Supporting Information

Contents

General Experimental procedure

Figure S1. HR-ESI-MS Full-spectrum (A) and zoom-spectrum (B) of compound **1**

Figure S2. ^{1}H NMR spectrum of compound **1** (800 MHz, pyridine-d_5)

Figure S3. ^{13}C NMR spectrum of compound **1** (200 MHz, pyridine-d_5)

Figure S4. ^{1}H-^{13}C HSQC spectrum of compound **1**

Figure S5. ^{1}H-^{13}C HMBC spectrum of compound **1**

Figure S6. ^{1}H-^{1}H ROESY spectrum of compound **1**

Figure S7. HSQC-TOSCY spectrum of compound **1**

Figure S8. HR-ESI-MS spectrum of compound **2**

Figure S9. ^{1}H NMR spectrum of compound **2** (500 MHz, pyridine-d_5)

Figure S10. ^{13}C NMR spectrum of compound **2** (125 MHz, pyridine-d_5)

Figure S11. ^{1}H-^{13}C HSQC spectrum of compound **2**

Figure S12. ^{1}H-^{13}C HMBC spectrum of compound **2**

Figure S13. ^{1}H-^{1}H ROESY spectrum of compound **2**

Figure S14. HR-ESI-MS spectrum of compound **3**

Figure S15. ^{1}H NMR spectrum of compound **3** (500 MHz, pyridine-d_5)

Figure S16. ^{13}C NMR spectrum of compound **3** (125 MHz, pyridine-d_5)

Figure S17. ^{1}H-^{13}C HSQC spectrum of compound **3**

Figure S18. ^{1}H-^{13}C HMBC spectrum of compound **3**

Figure S19. ^{1}H-^{1}H NOESY spectrum of compound **3**

Figure S20. A: Extracted ion chromatograms (EICs) of derivatized glucose and hydrolyzed samples in negative mode (mass range of *m/z* 444.50–445.50, D-glucose: t_R 11.0 min), B: Full MS spectrum of derivatized glucose and hydrolyzed samples.

Figure S21. A: Extracted ion chromatograms (EICs) of derivatized rhamnose and hydrolyzed samples in negative mode (mass range of *m/z* 428.50–429.50, L-rhamnose: t_R 18.31 min), B: Full MS spectrum of derivatized rhamnose and hydrolyzed samples.

Figure S22. Extracted ion chromatograms (EICs) of derivatized pentose and hydrolyzed samples in negative mode (mass range of *m/z* 414.50–415.50, D-apiose: t_R 16.82 min; L-arabinose: t_R 9.01 min; L-xylose: t_R 12.22 min)

Figure S23. Full MS spectrum of derivatized apiose and hydrolyzed samples.

Figure S24. Full MS spectrum of derivatized arabinose and hydrolyzed samples.

Figure S25. Full MS spectrum of derivatized xylose and hydrolyzed samples.

Scheme S1. Isolation scheme of compounds **1**–**6** from the 95% EtOH extract of *A. koraiensis* leaves

Table S1. ^{1}H and ^{13}C NMR spectroscopic data of compound **4** (δ in ppm, pyridine-d_5, 500 and 125 MHz)

Table S2. ^{1}H and ^{13}C NMR spectroscopic data of compound **5** (δ in ppm, pyridine-d_5, 500 and 125 MHz)

Table S3. ^{1}H and ^{13}C NMR spectroscopic data of compound **6** (δ in ppm, pyridine-d_5, 500 and 125 MHz)

General Experimental procedure

MPA 100 (Stanford research systems, Sunnyvale, CA, USA) was used to measure melting points in open capillary tubes. JEOL (JEOL, Tokyo, Japan) 500 MHz was used for obtaining NMR spectra. HR-Mass spectra were obtained by a Q-TOF micro mass spectrometer (Waters, Milford, Massachusetts, USA). TLC analyses were performed on Silica gel 60 F_{254} (Merck, Kenilworth, MA, USA) and RP-18 F_{254S} (Merck) plates. Compounds were visualized by dipping plates into 20% (v/v) H_2SO_4 reagent (Samchun) and then heated at 110°C for 5-10 min. Agilent Cary 630 FTIR (Agilent Technologies, Santa Clara, CA, USA) was applied to obtain IR spectrum. Sephadex LH-20 (Amersham Pharmacia Biotech, Buckinghamshire, United Kingdom), Silica gel (Merck 60A, 230-400 mesh ASTM), Diaion HP-20 (Mitsubishi, Tokyo, Japan), and reversed-phase silica gel (YMC Co., ODS-A 12 nm S-150 μm) were used for column chromatography. Pre-packed cartridges with Redi Sep-Silica (12 g, 24 g, 40 g, Teledyne Isco) and Redi Sep-C18 (13 g, 26 g, 43 g, 130 g, Teledyne Isco) were used for flash chromatography. Flash chromatography was performed using the flash purification system (Combi Flash Rf, Teledyne Isco). HPLC was performed using Waters purification system (1525 pump, PDA 1996 detector) with Gemini NX-C18 110A column (250 × 21.2mm i.d. 5 μm, Phenomenex, Torrance, CA, USA). Before chromatographic separations, all solvents used for this study were distilled.

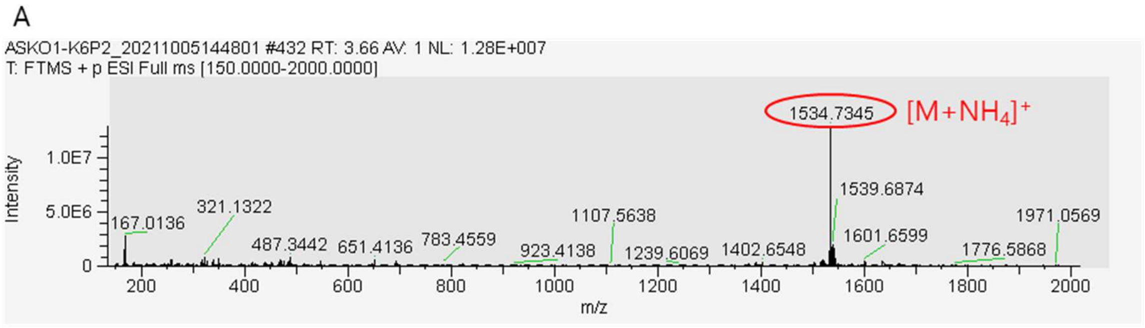

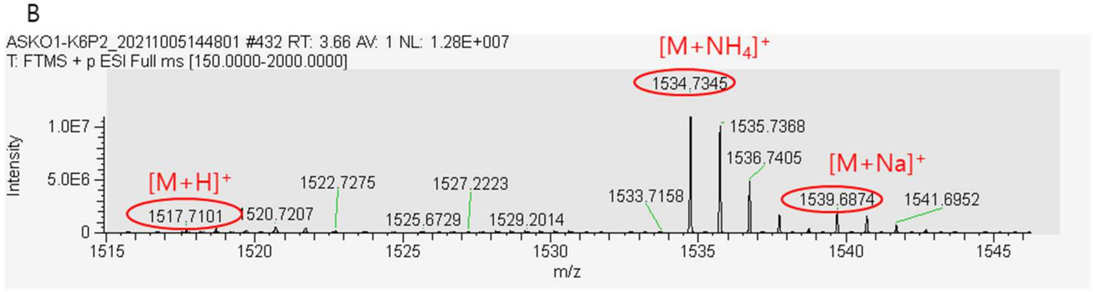

Figure S1. HR-ESI-MS Full-spectrum (A) and zoom-spectrum (B) of compound **1**

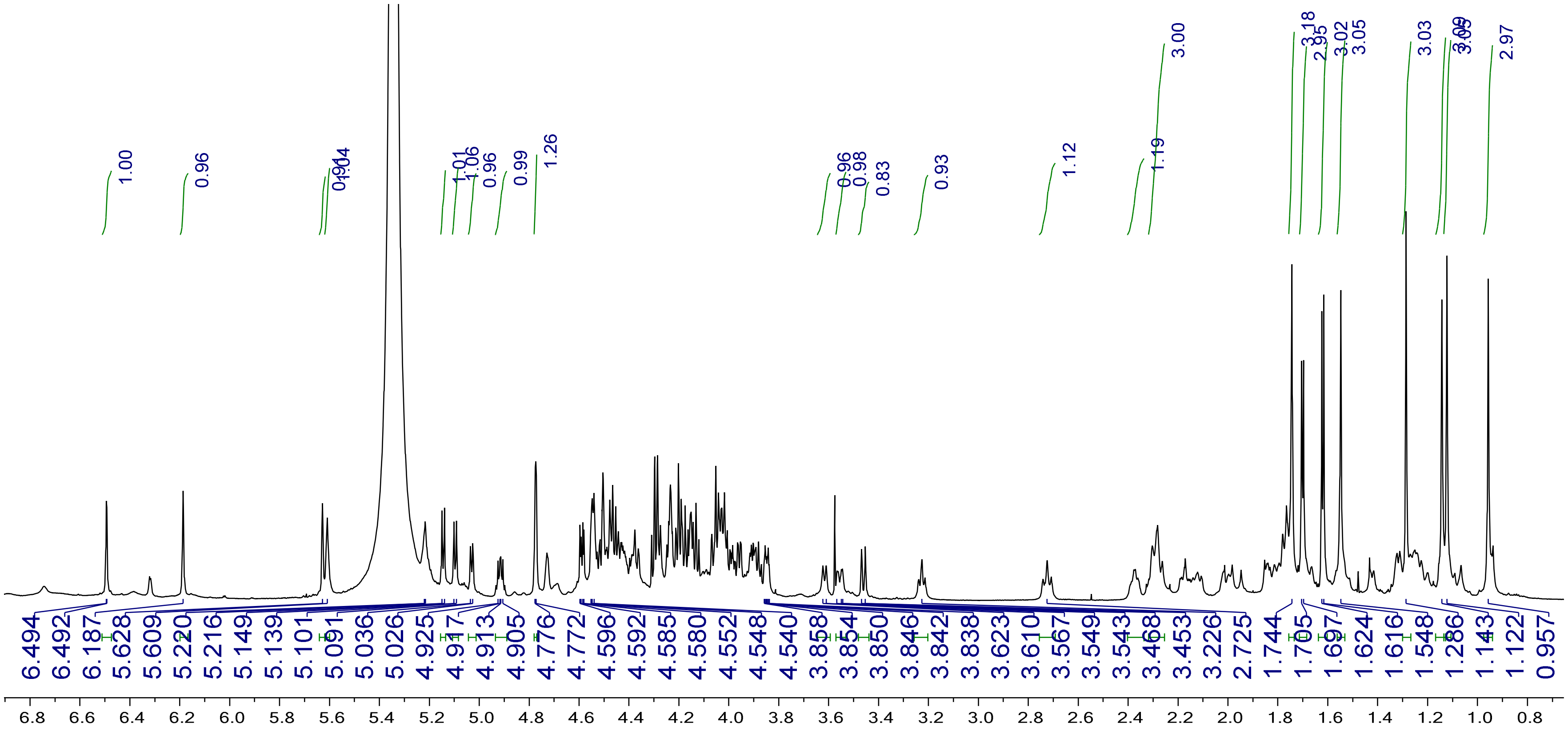

Figure S2. ^{1}H NMR spectrum of compound **1** (800 MHz, pyridine-d_5)

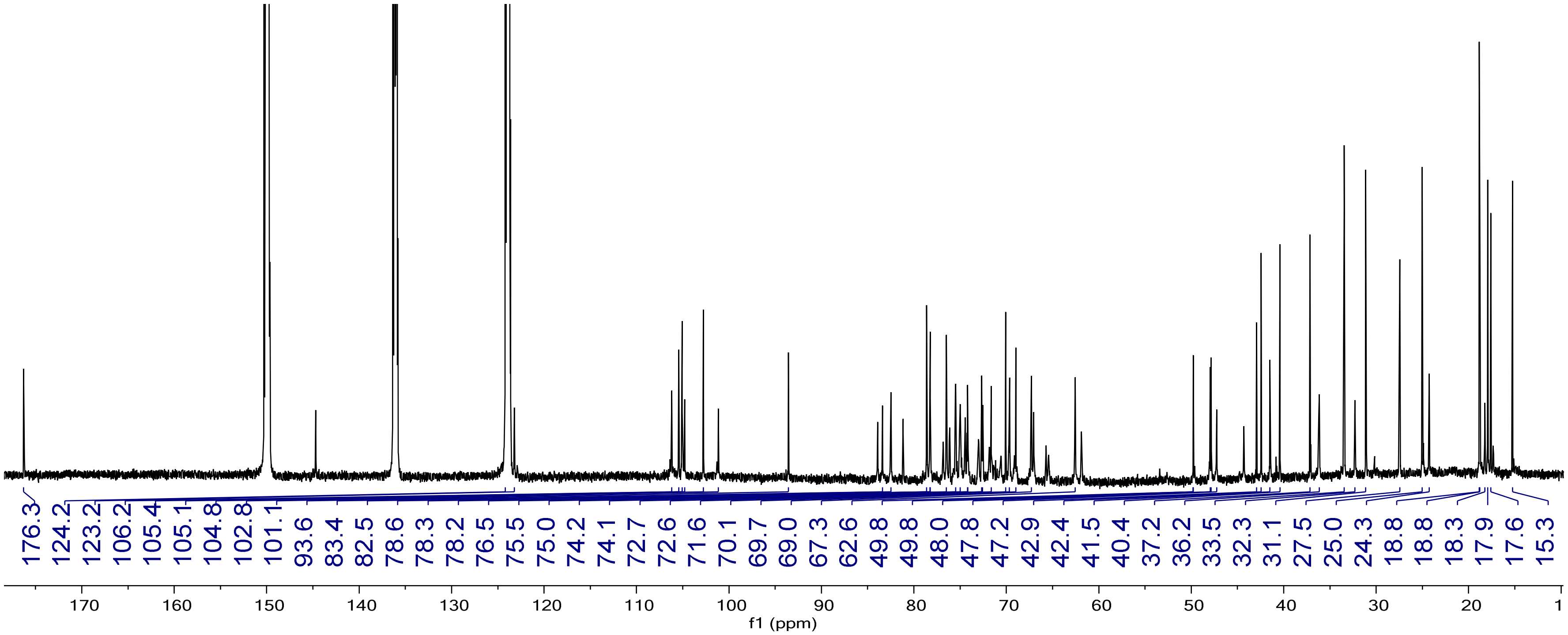

Figure S3. ^{13}C NMR spectrum of compound **1** (200 MHz, pyridine-d_5)

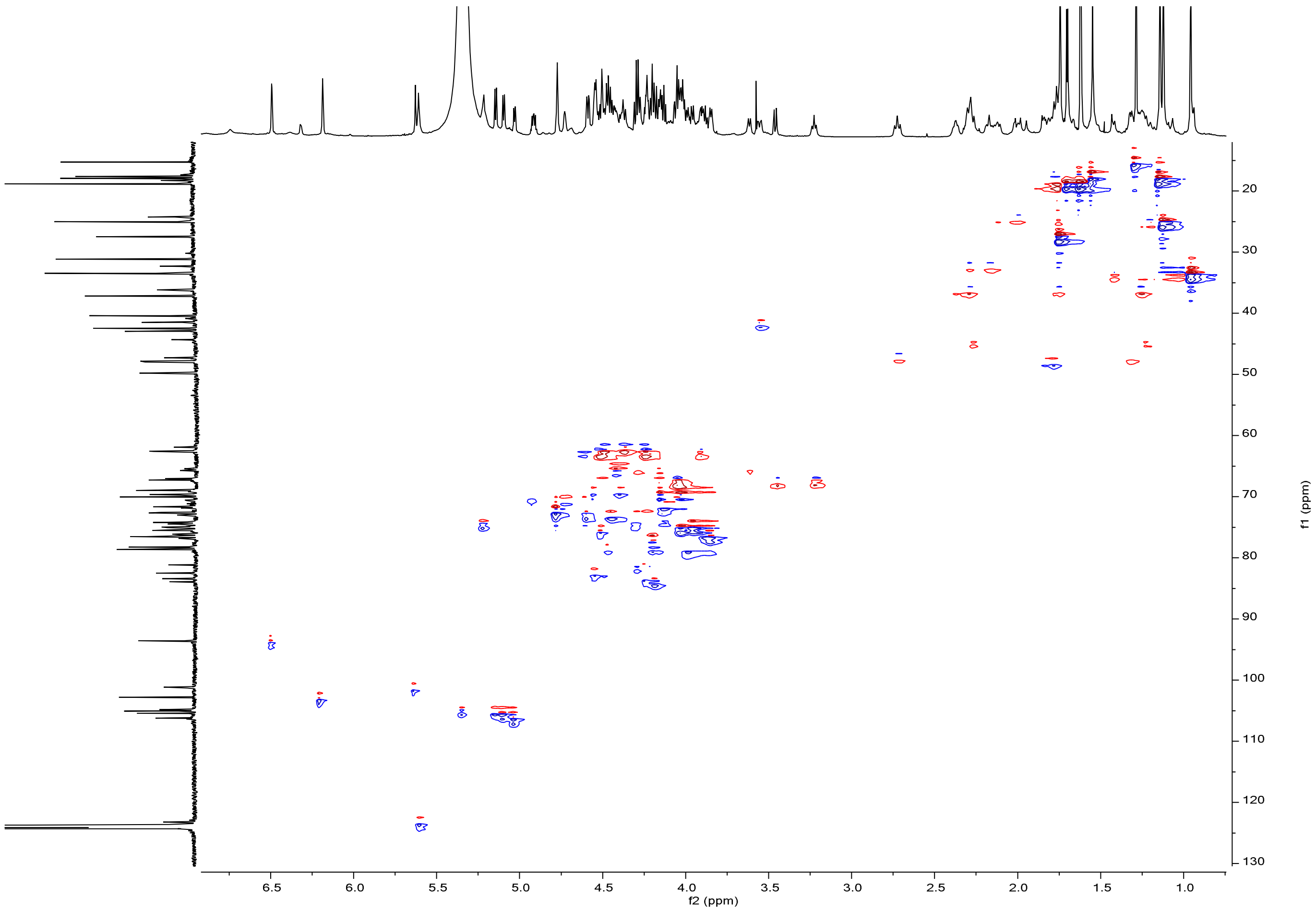

Figure S4. ^{1}H-^{13}C HSQC spectrum of compound **1**

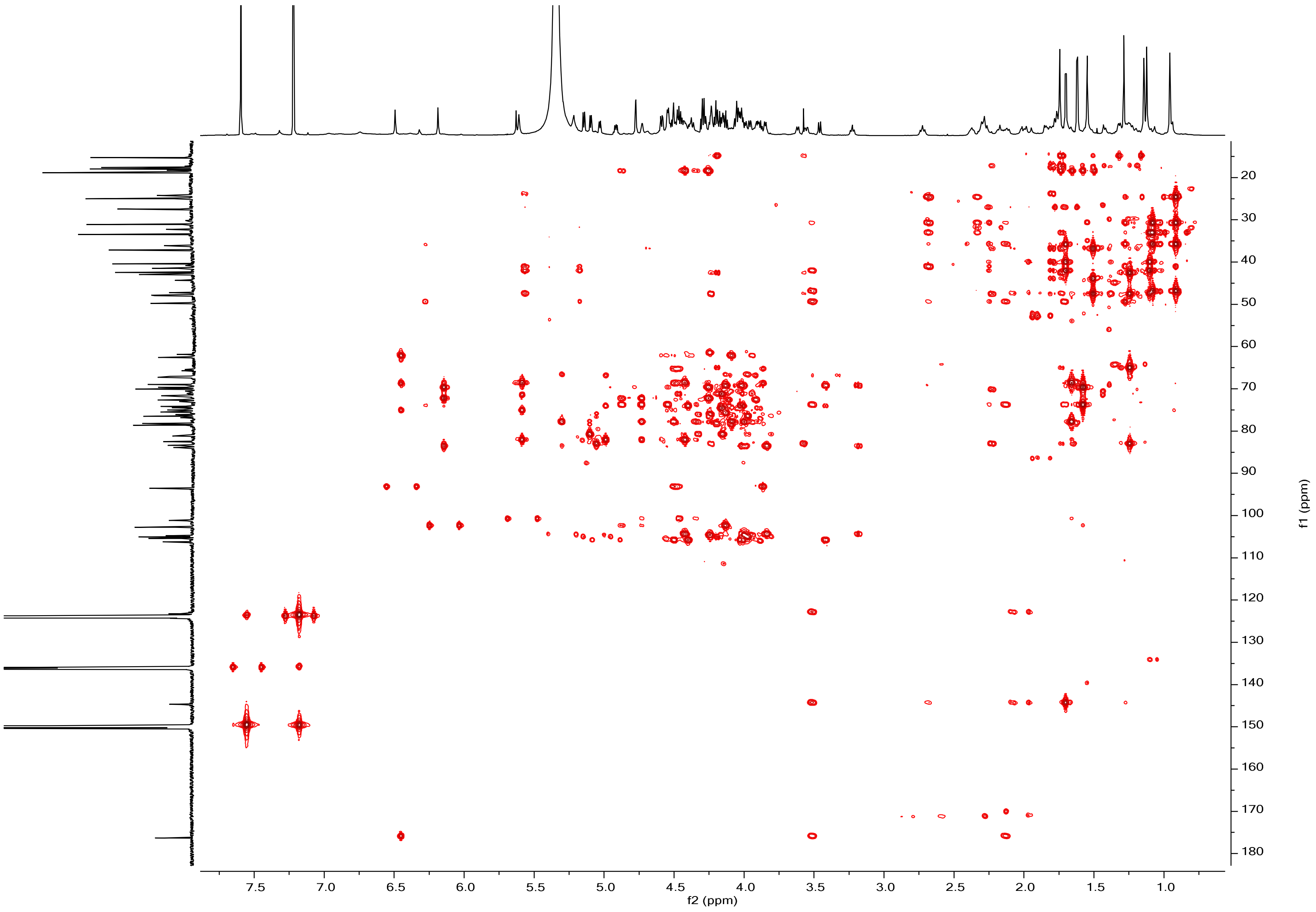

Figure S5. ^{1}H-^{13}C HMBC spectrum of compound **1**

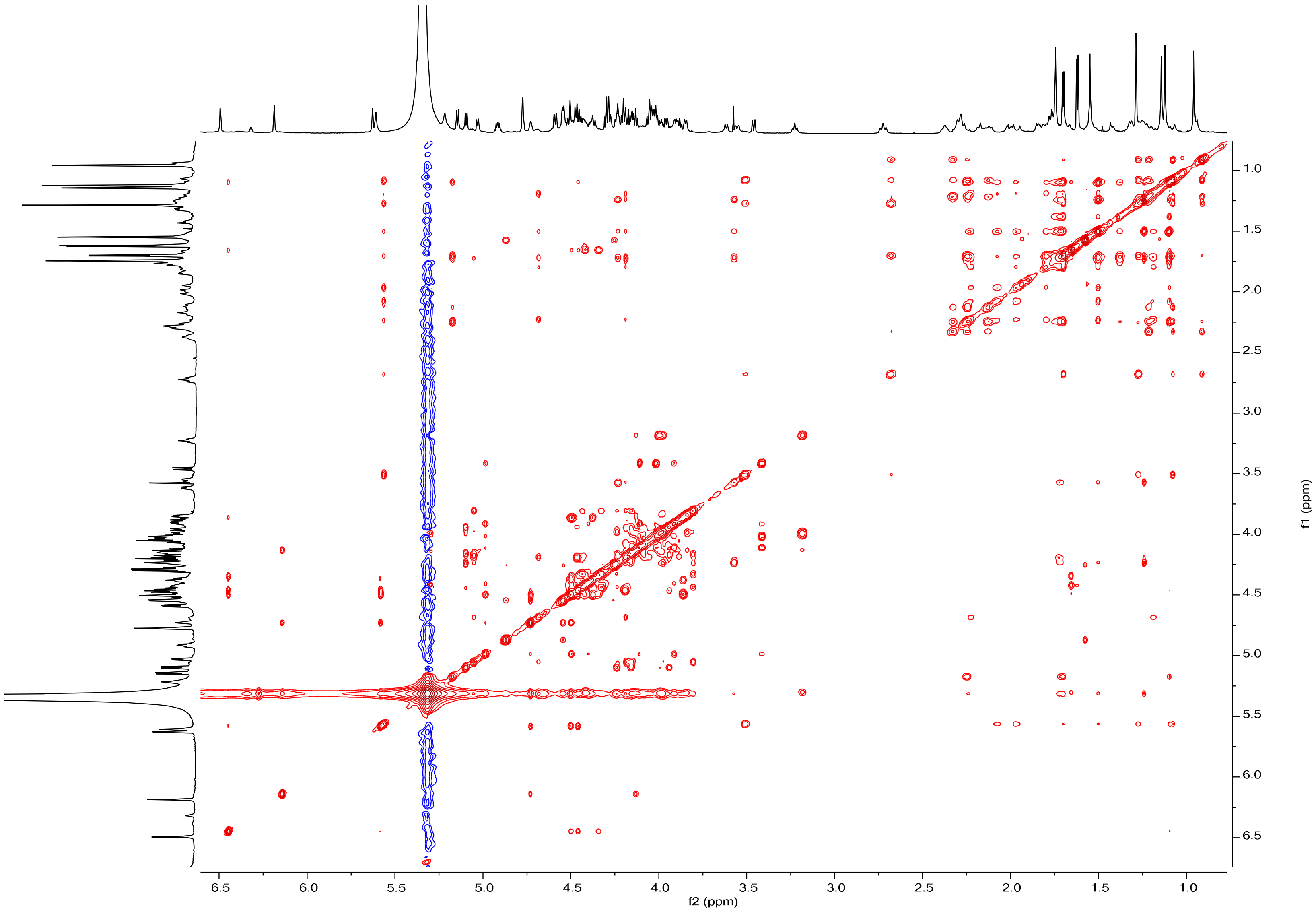

Figure S6. ^1H-^1H ROESY spectrum of compound **1**

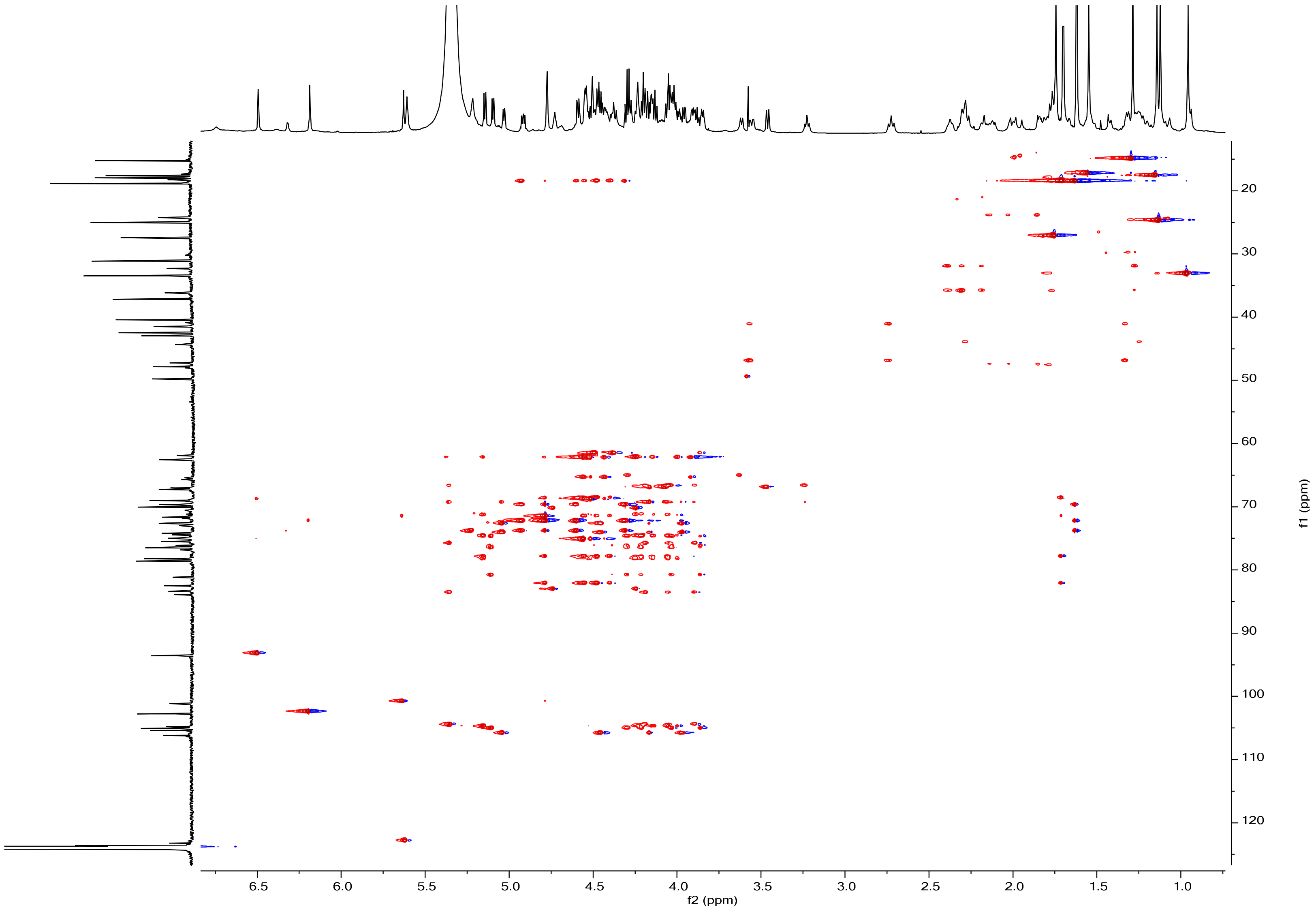

Figure S7. HSQC-TOSCY spectrum of compound **1**

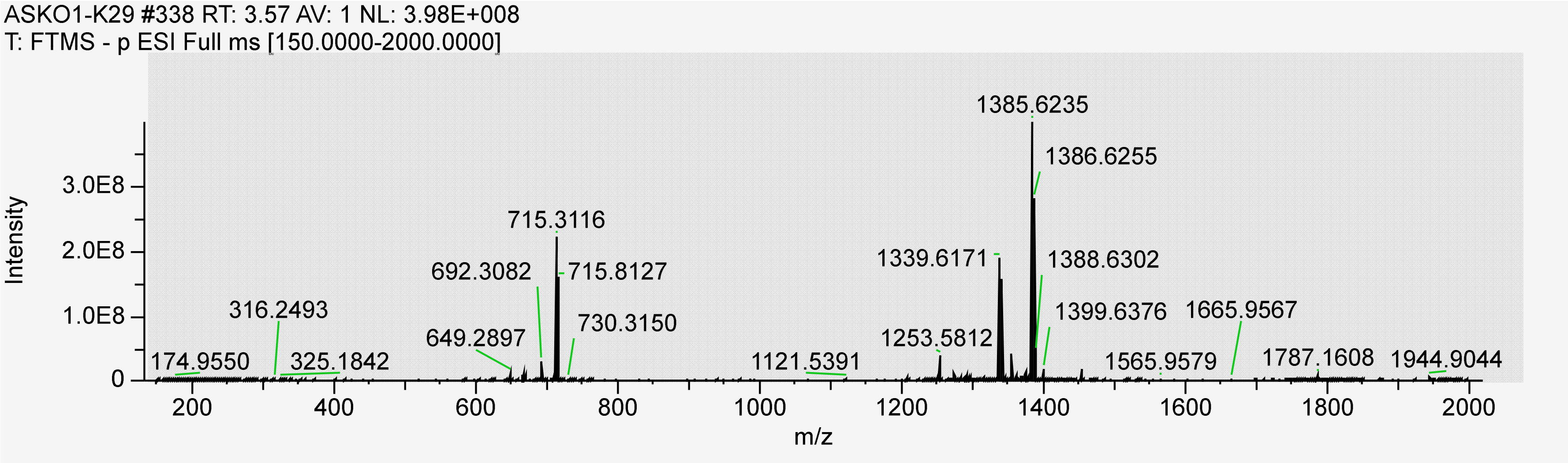

Figure S8. HR-ESI-MS spectrum of compound **2**

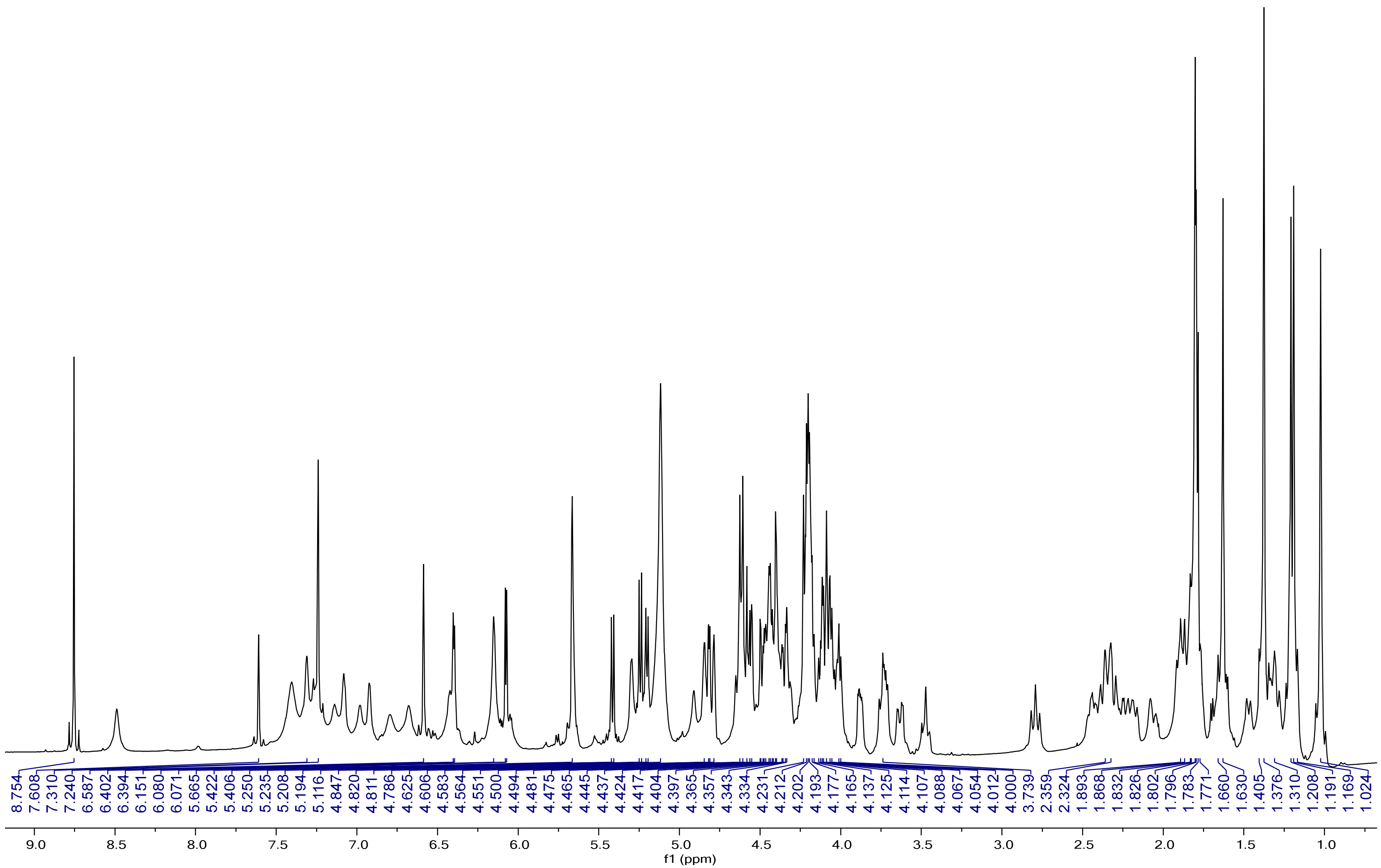

Figure S9. ^{1}H NMR spectrum of compound **2** (500 MHz, pyridine-d_5)

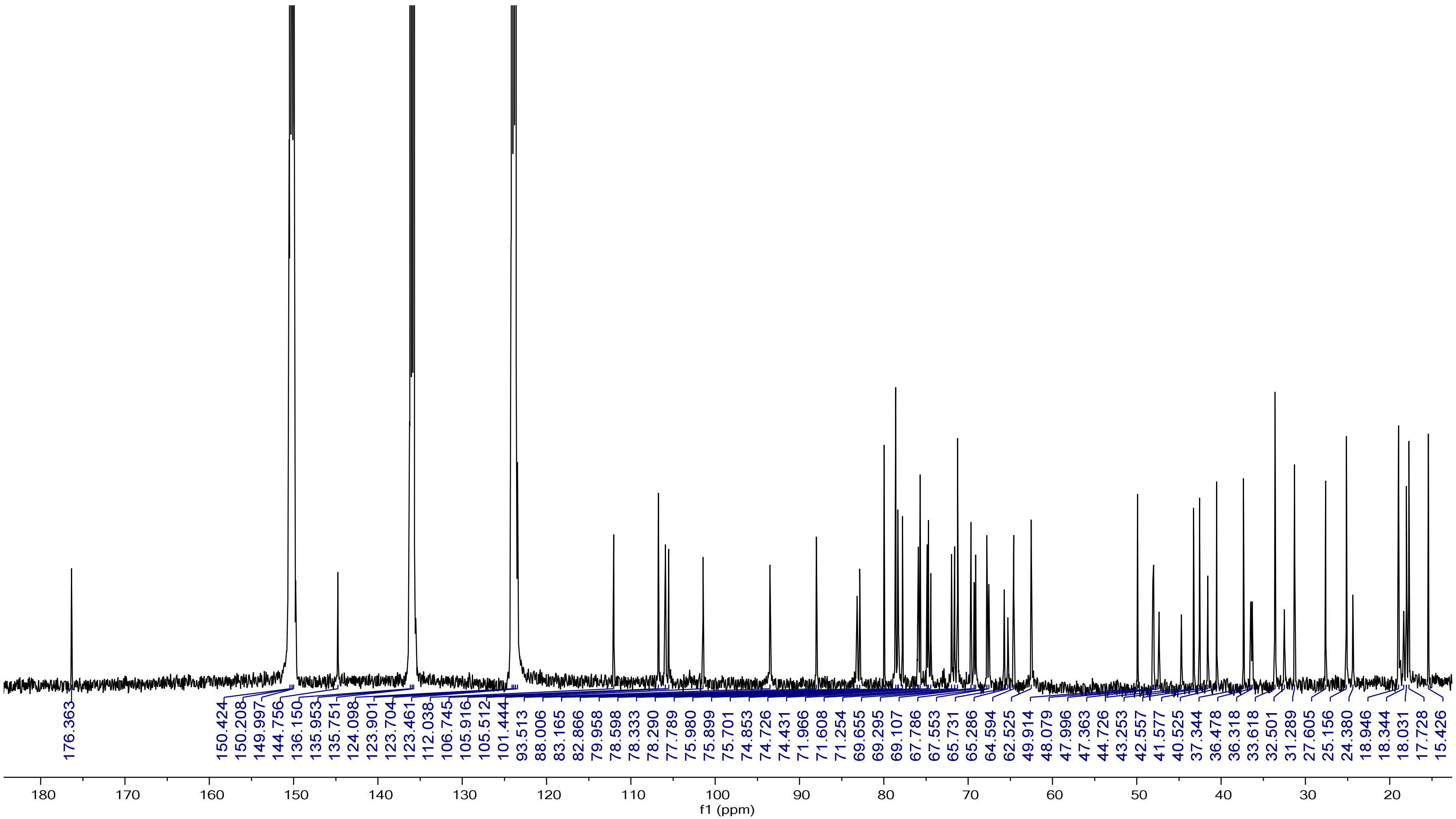

Figure S10. ^{13}C NMR spectrum of compound **2** (125 MHz, pyridine-d_5)

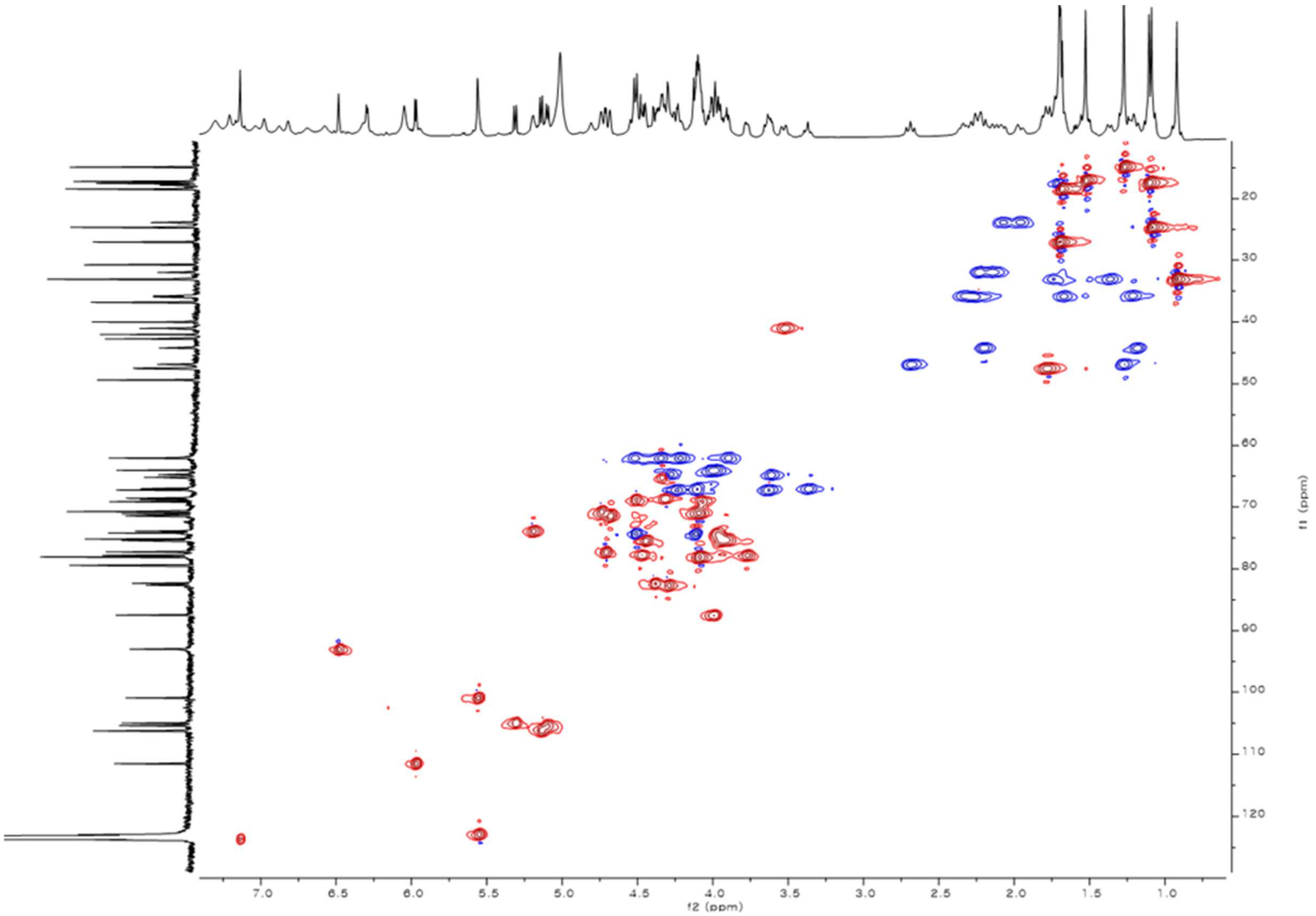

Figure S11. ^{1}H-^{13}C HSQC spectrum of compound **2**

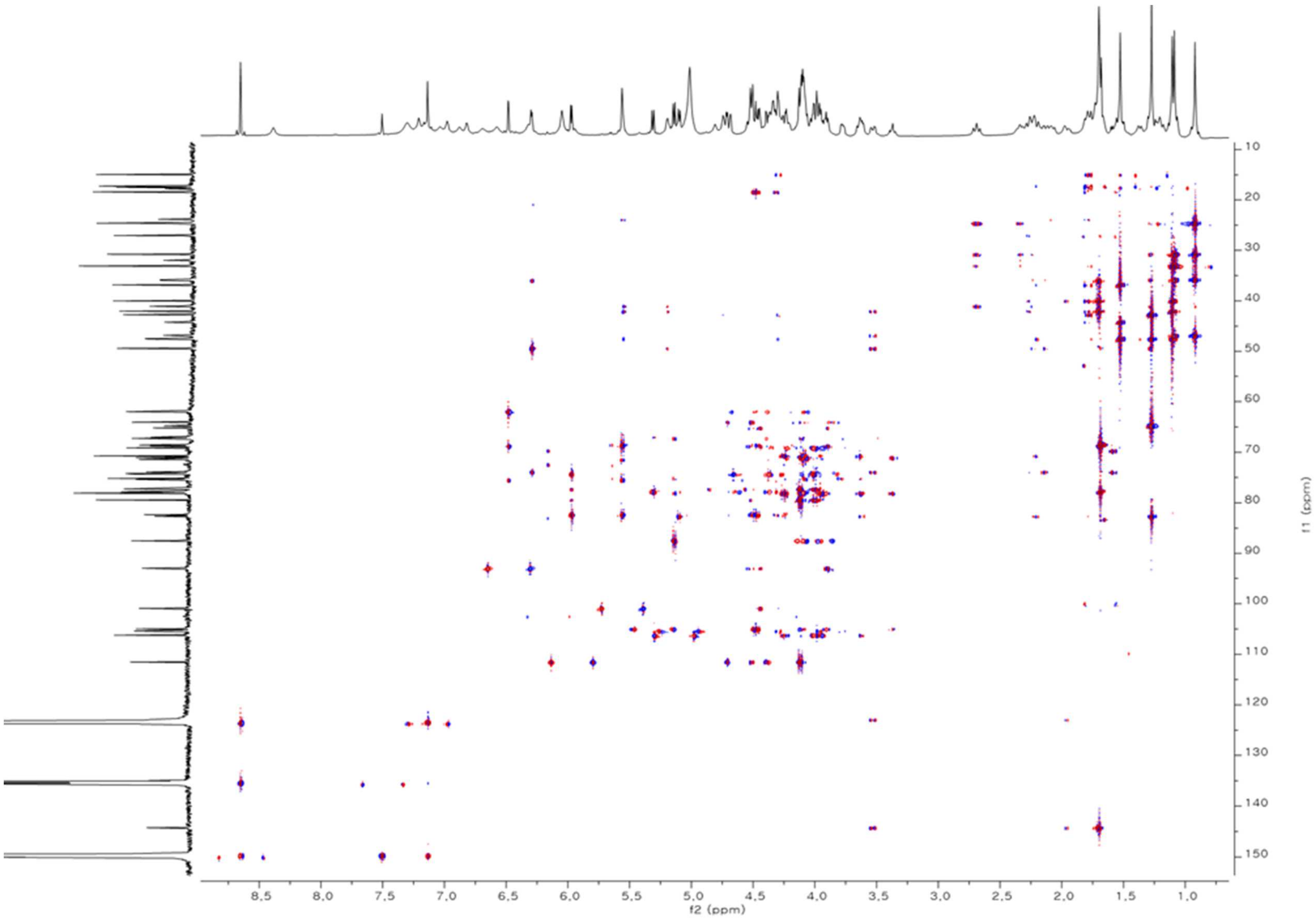

Figure S12. ^{1}H-^{13}C HMBC spectrum of compound **2**

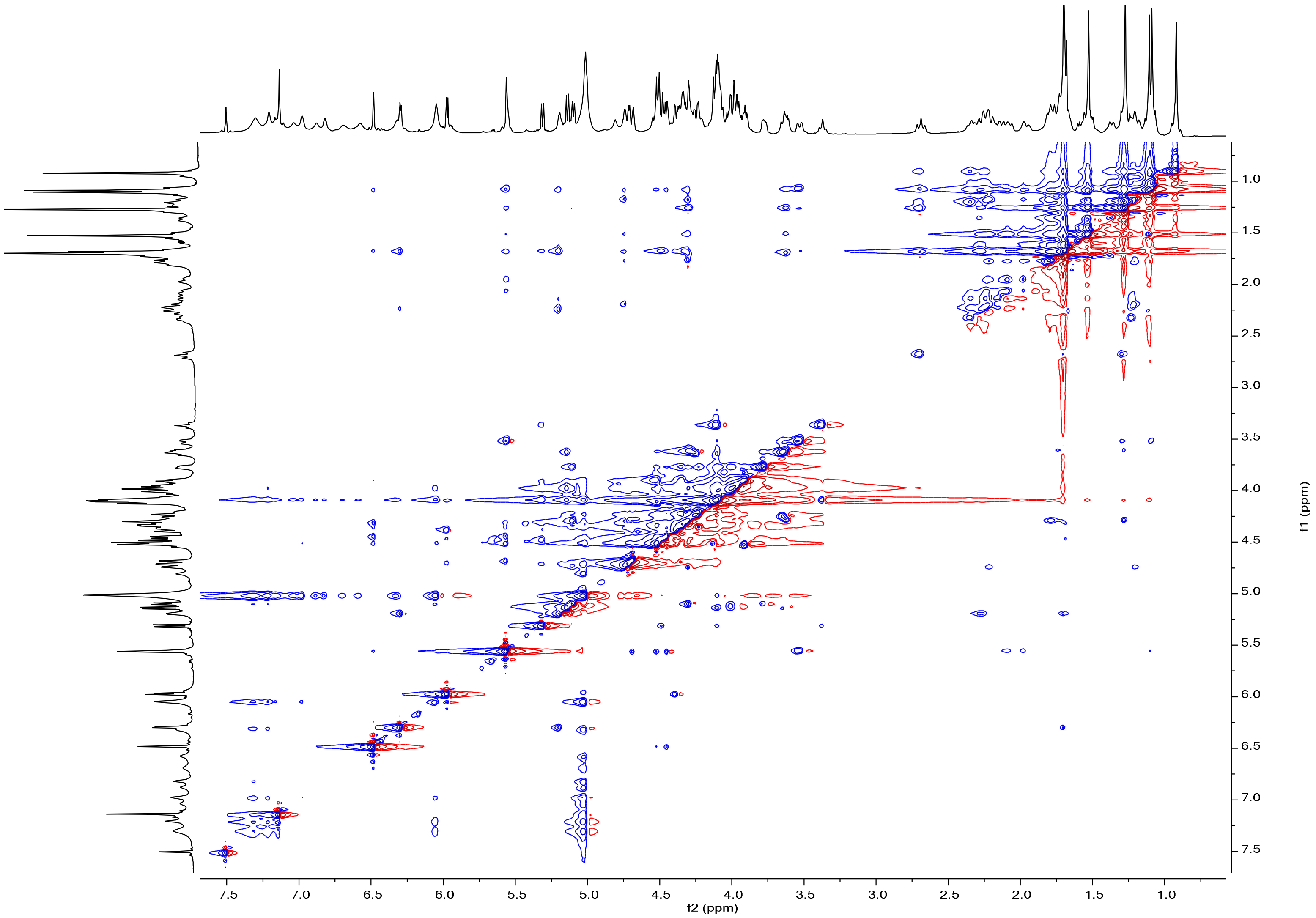

Figure S13. ^{1}H-^{1}H ROESY spectrum of compound **2**

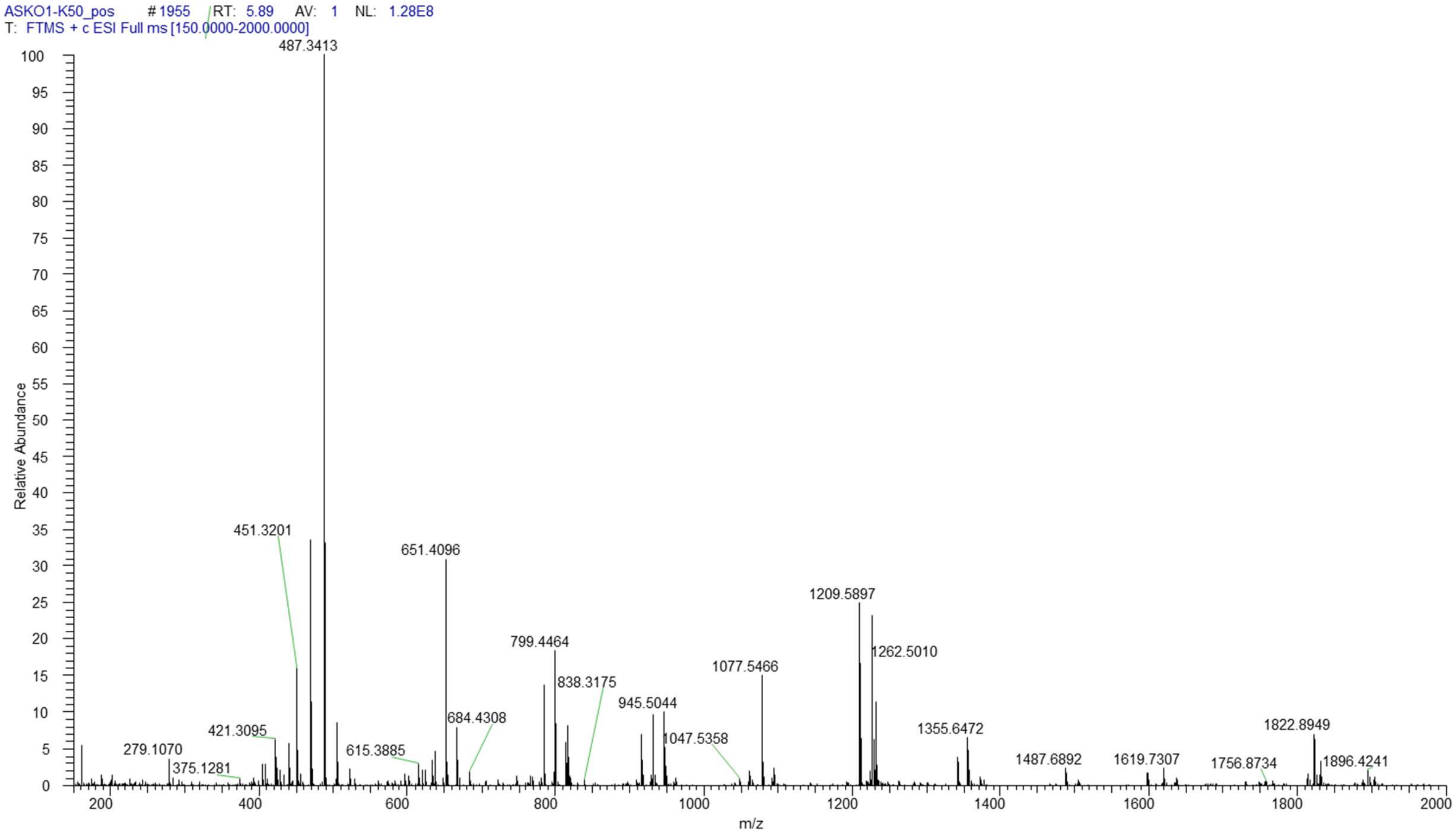

Figure S14. HR-ESI-MS spectrum of compound **3**

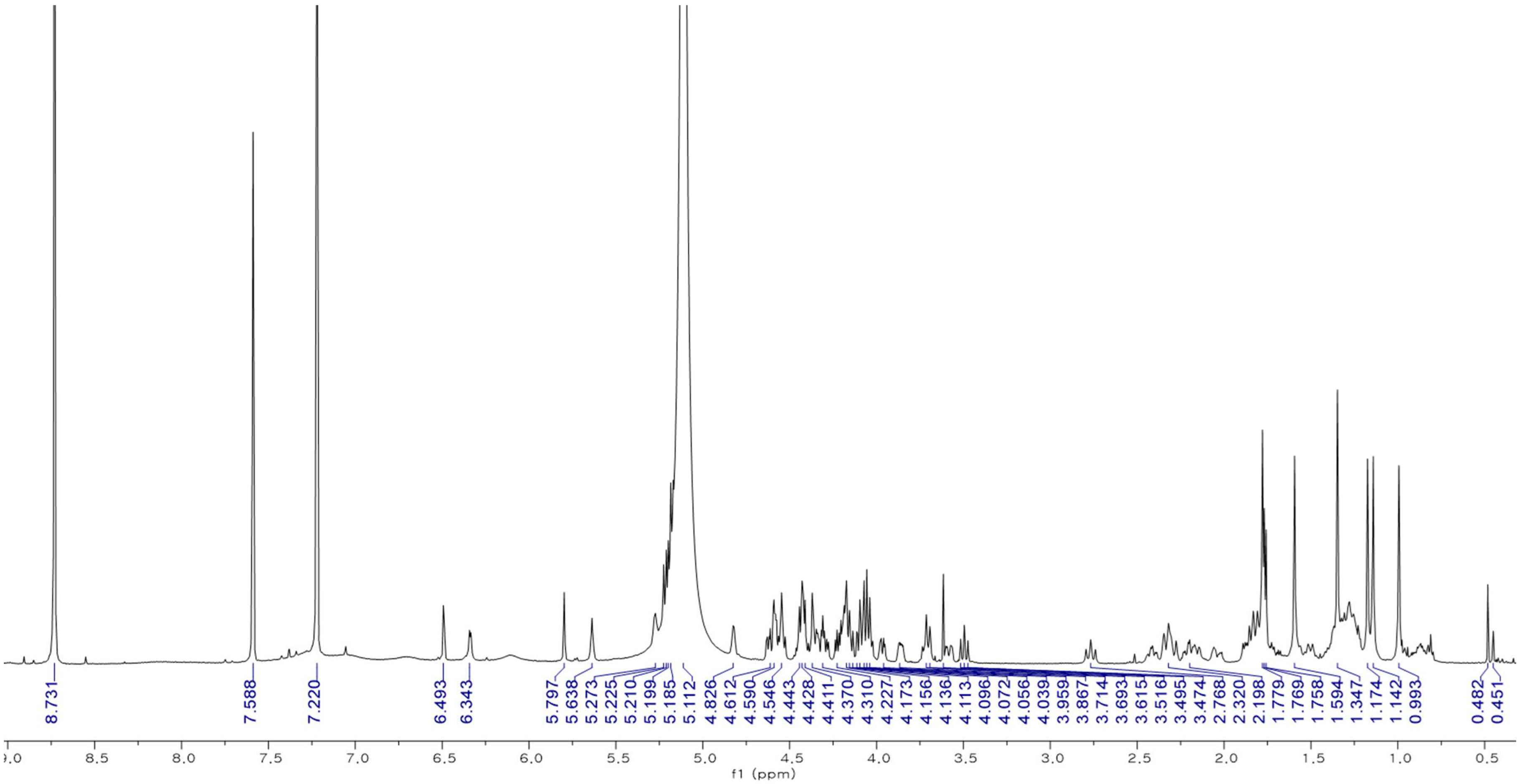

Figure S15. ^{1}H NMR spectrum of compound **3** (500 MHz, pyridine-d_5)

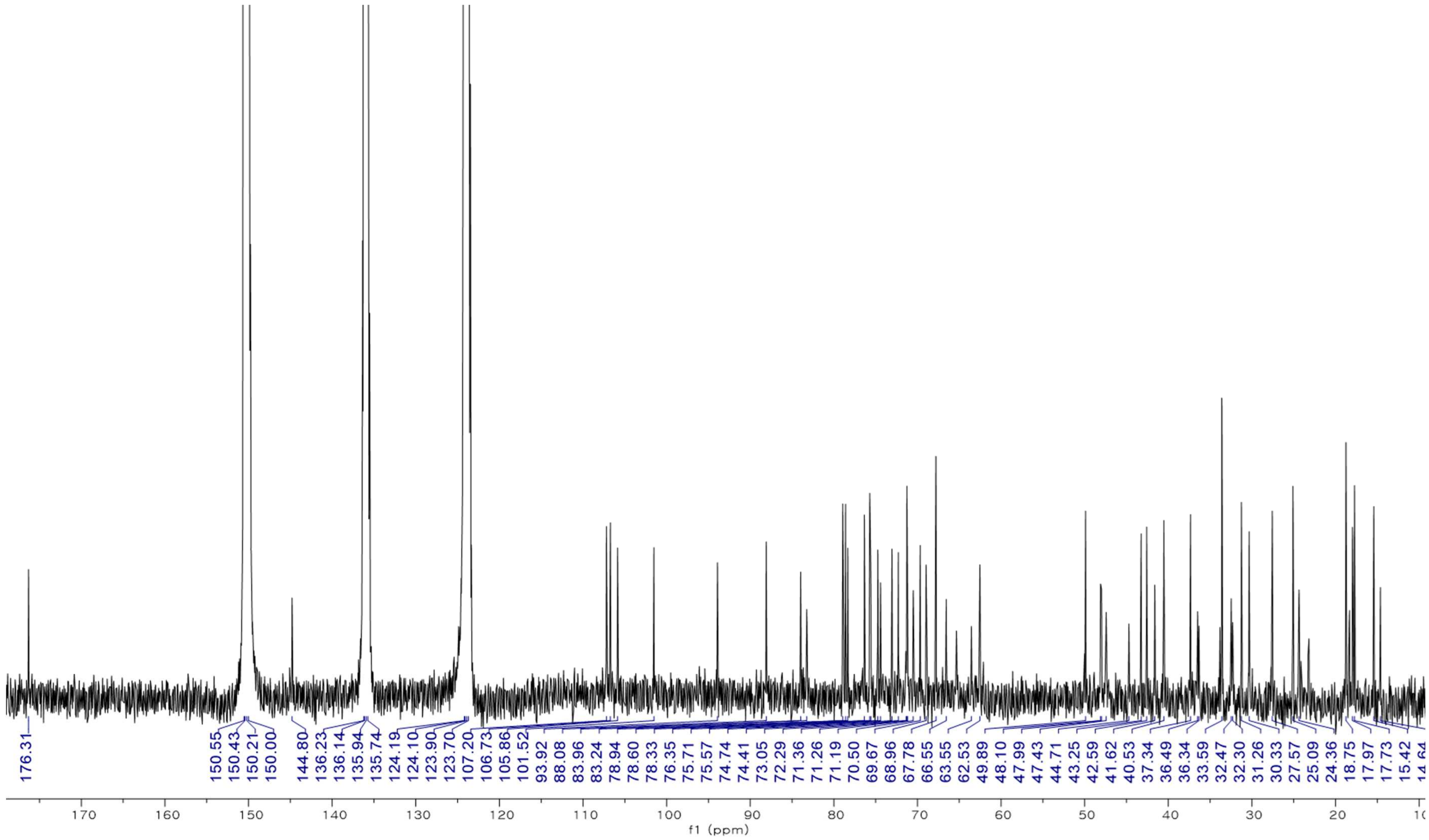

Figure S16. ^{13}C NMR spectrum of compound **3** (125 MHz, pyridine-d_5)

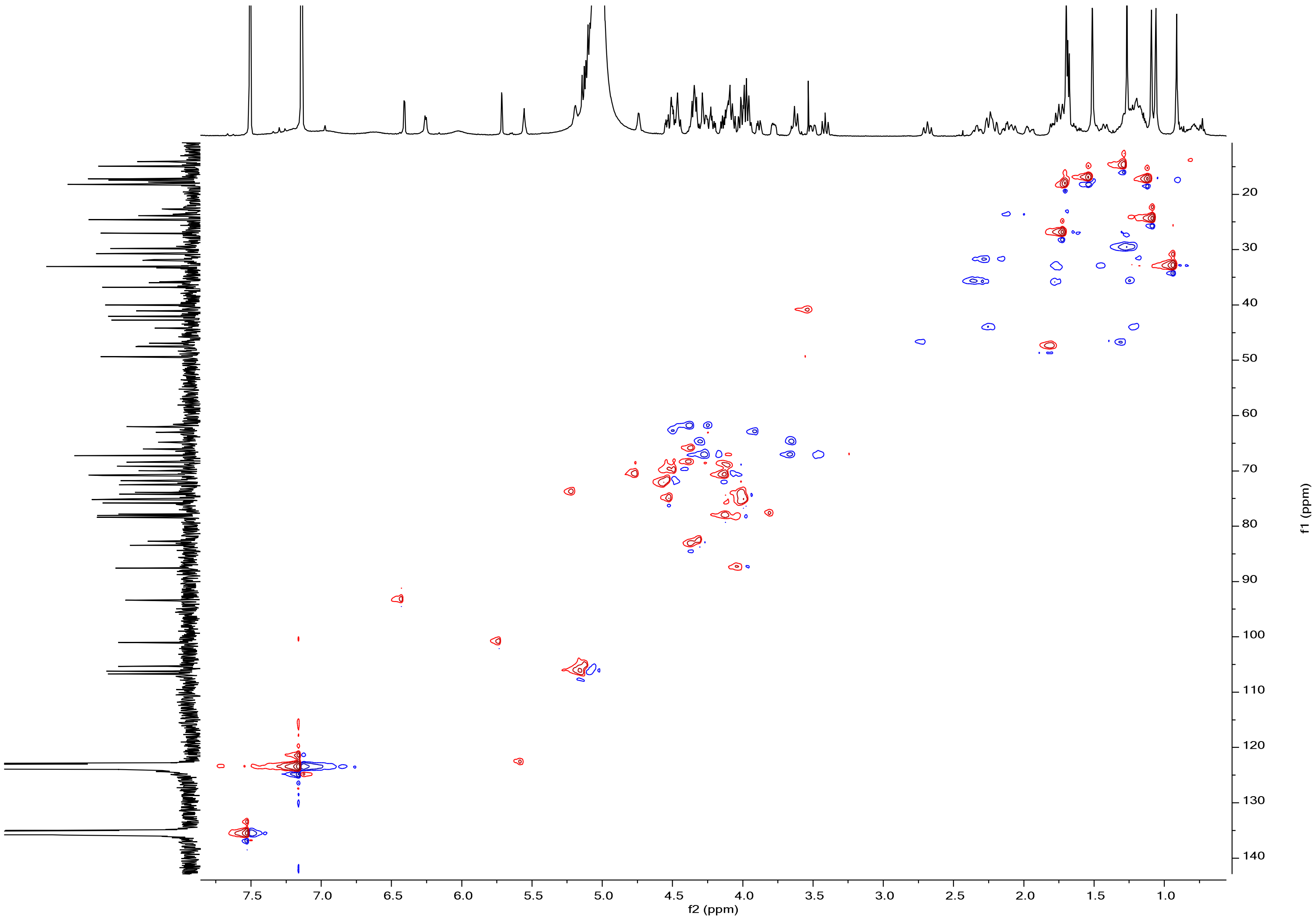

Figure S17. ^{1}H-^{13}C HSQC spectrum of compound **3**

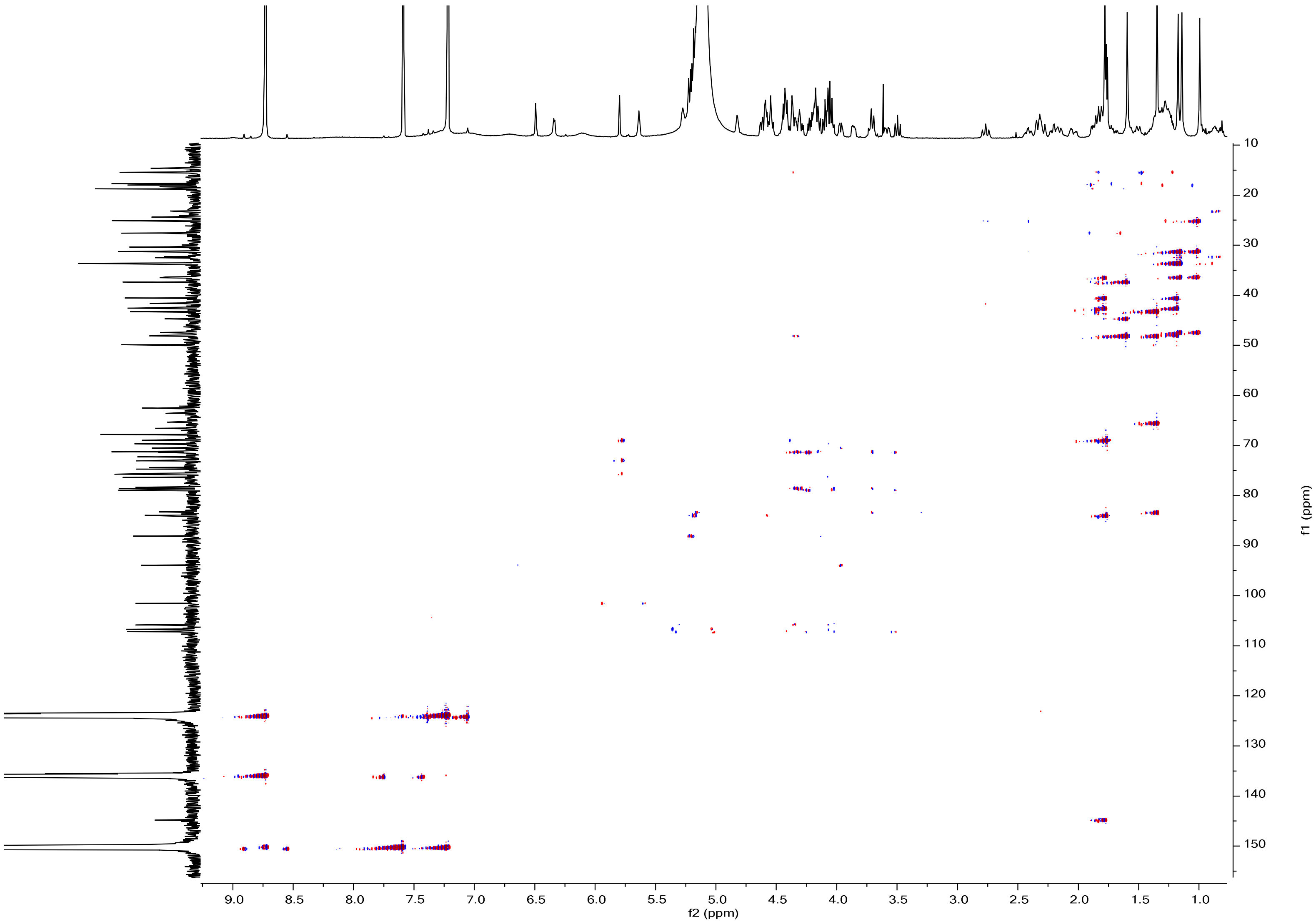

Figure S18. ^{1}H-^{13}C HMBC spectrum of compound **3**

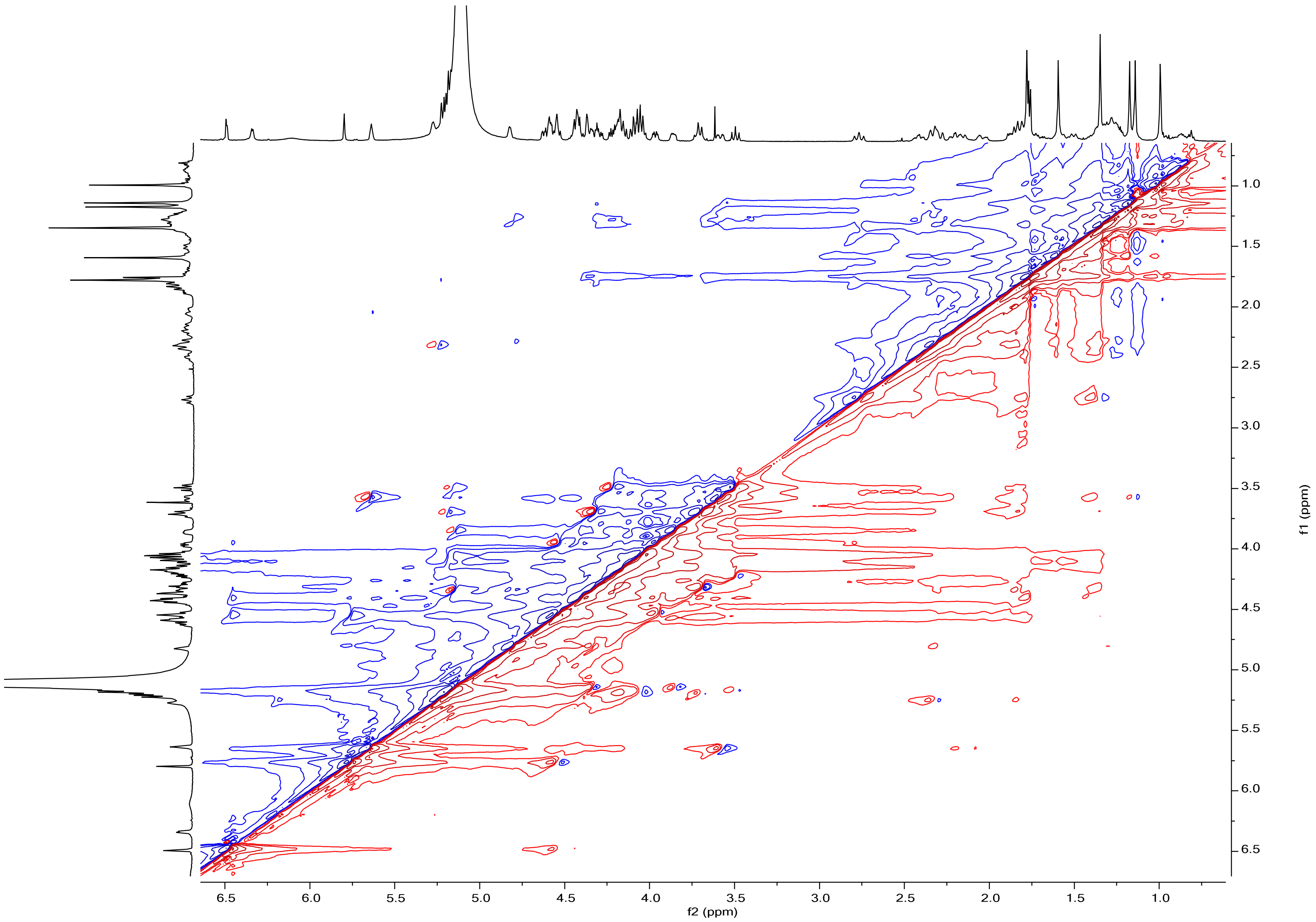

Figure S19. ^{1}H-^{1}H NOESY spectrum of compound **3**

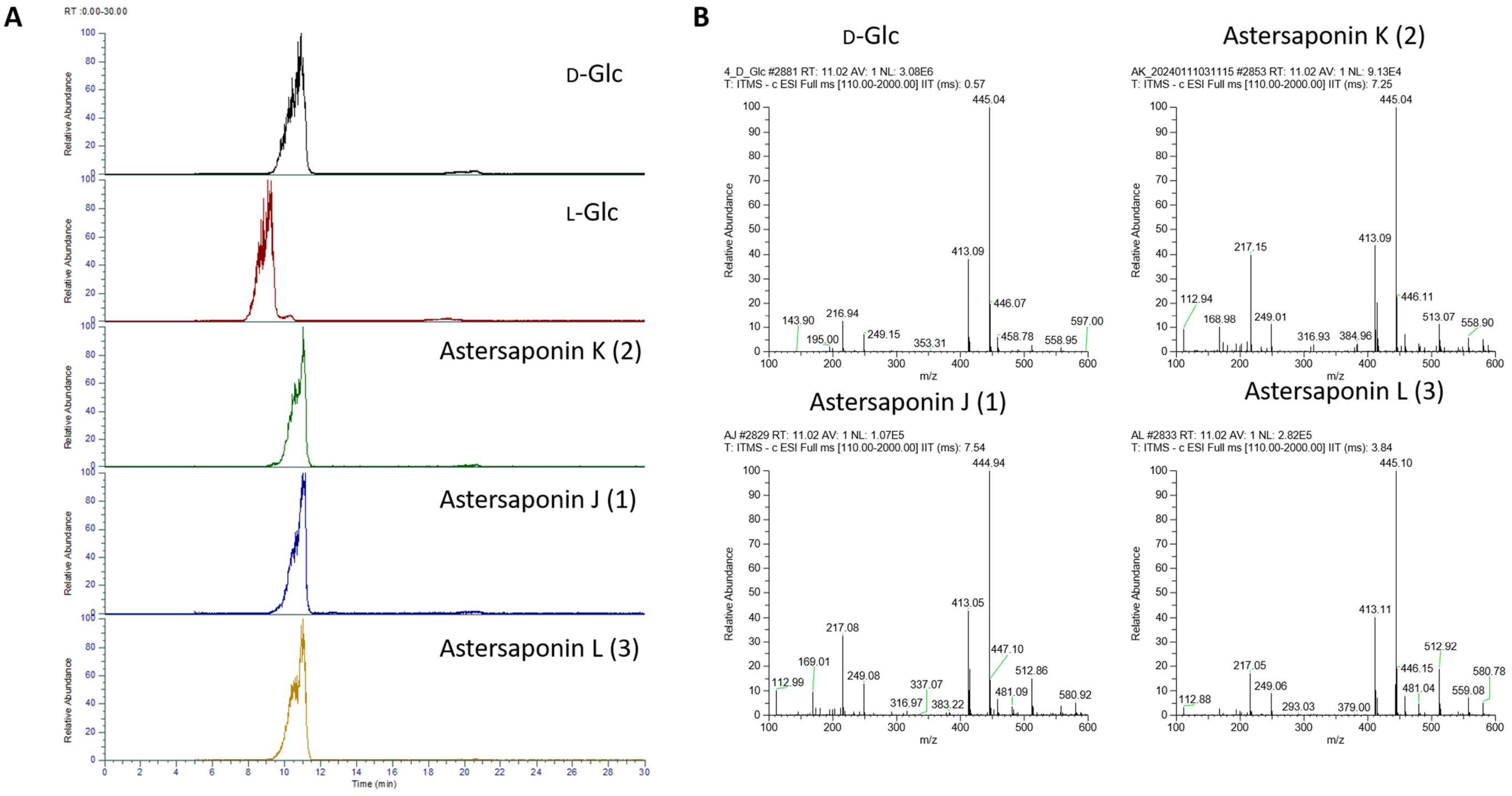

Figure S20. A: Extracted ion chromatograms (EICs) of derivatized glucose and hydrolyzed samples in negative mode (mass range of *m/z* 444.50–445.50, D-glucose: t_R 11.0 min), B: Full MS spectrum of derivatized glucose and hydrolyzed samples.

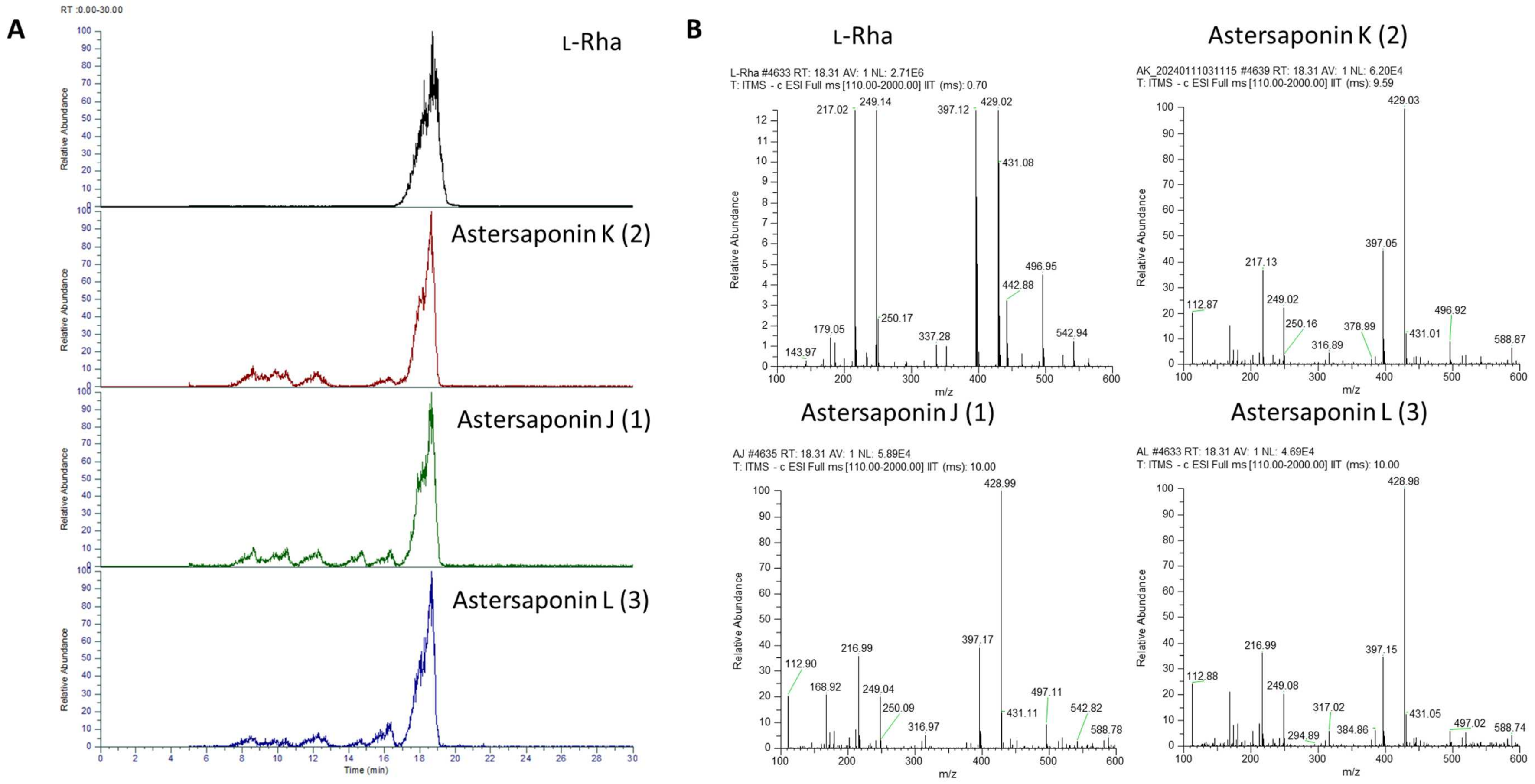

Figure S21. A: Extracted ion chromatograms (EICs) of derivatized rhamnose and hydrolyzed samples in negative mode (mass range of *m/z* 428.50–429.50, L-rhamnose: t_R 18.31 min), B: Full MS spectrum of derivatized rhamnose and hydrolyzed samples.

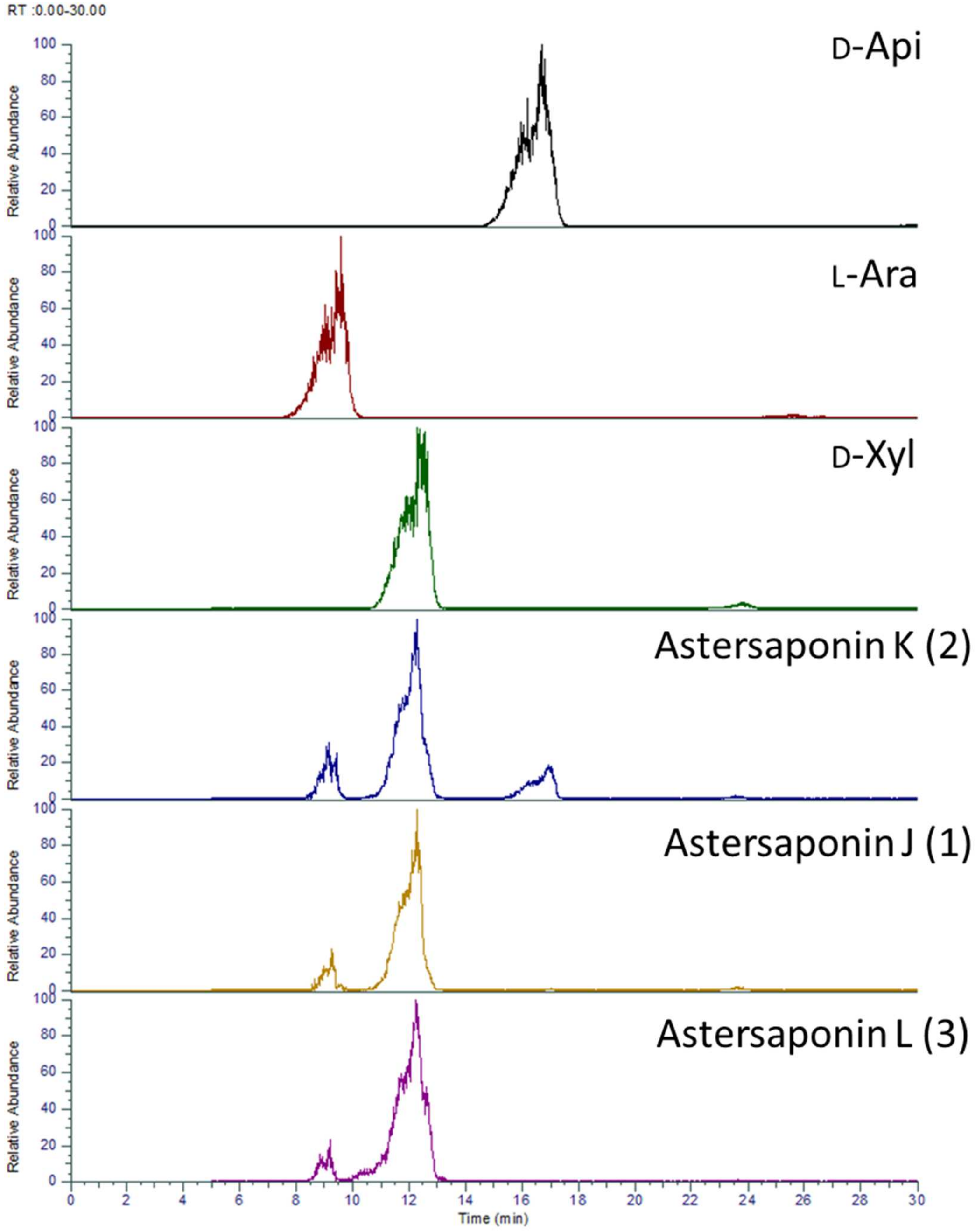

Figure S22. Extracted ion chromatograms (EICs) of derivatized pentose and hydrolyzed samples in negative mode (mass range of *m/z* 414.50–415.50, D-apiose: t_R 16.82 min; L-arabinose: t_R 9.01 min; L-xylose: t_R 12.22 min)

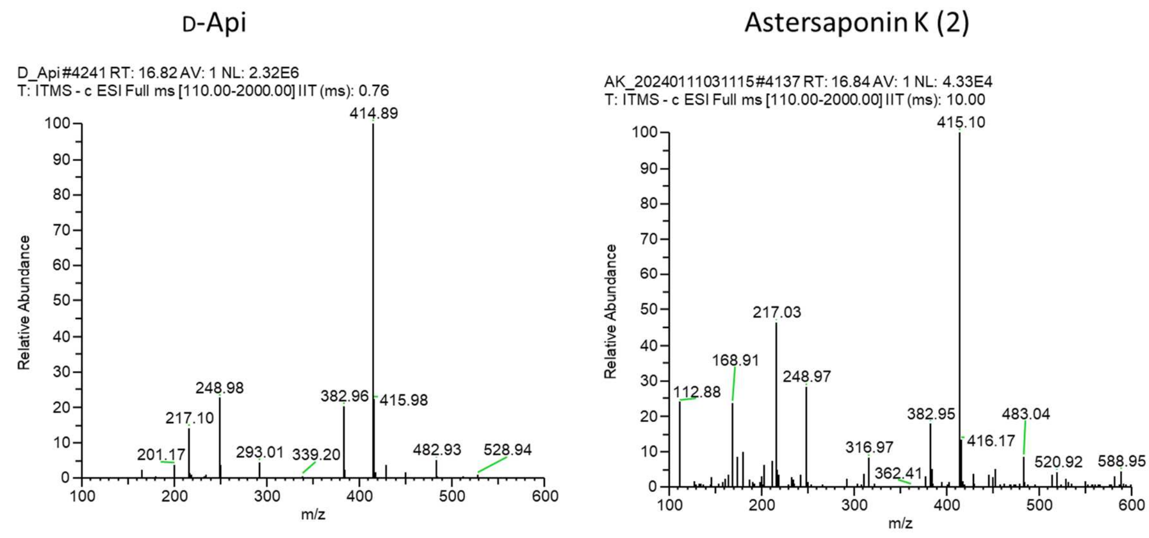

Figure S23. Full MS spectrum of derivatized apiose and hydrolyzed samples.

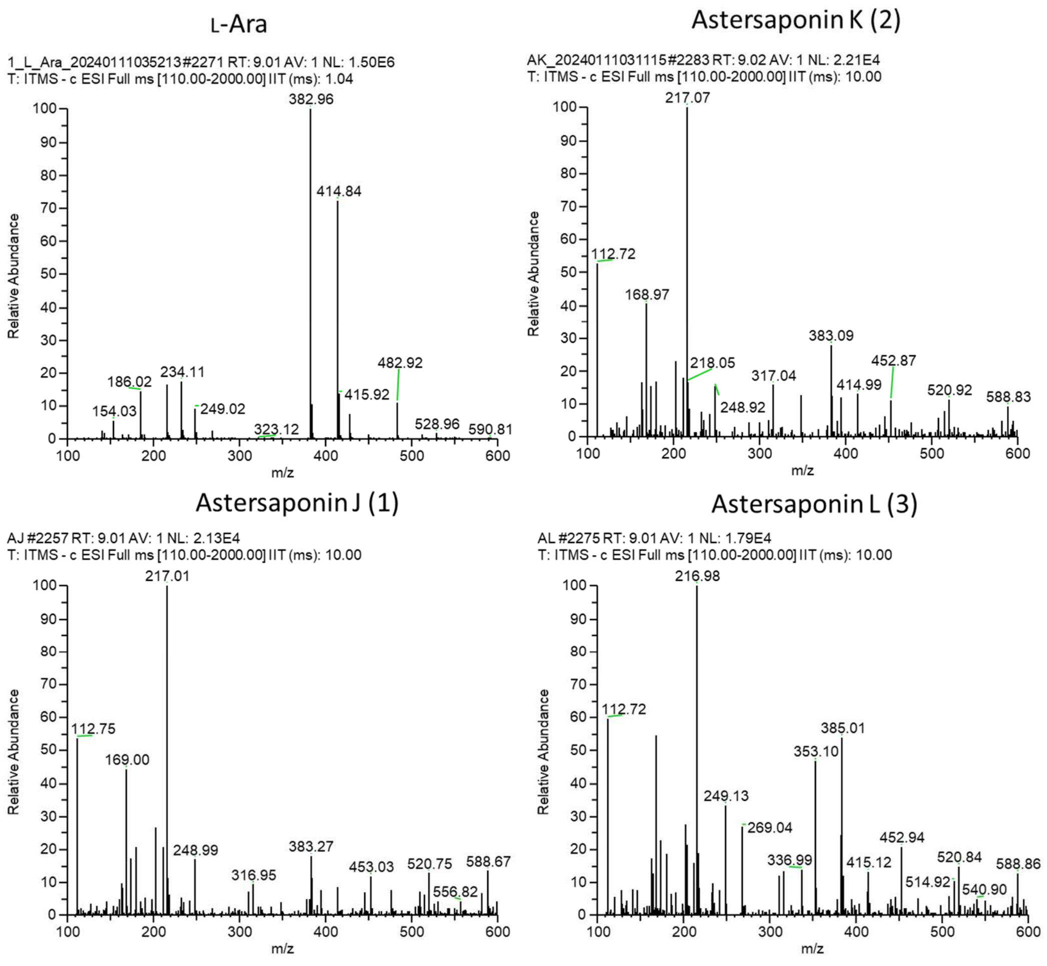

Figure S24. Full MS spectrum of derivatized arabinose and hydrolyzed samples.

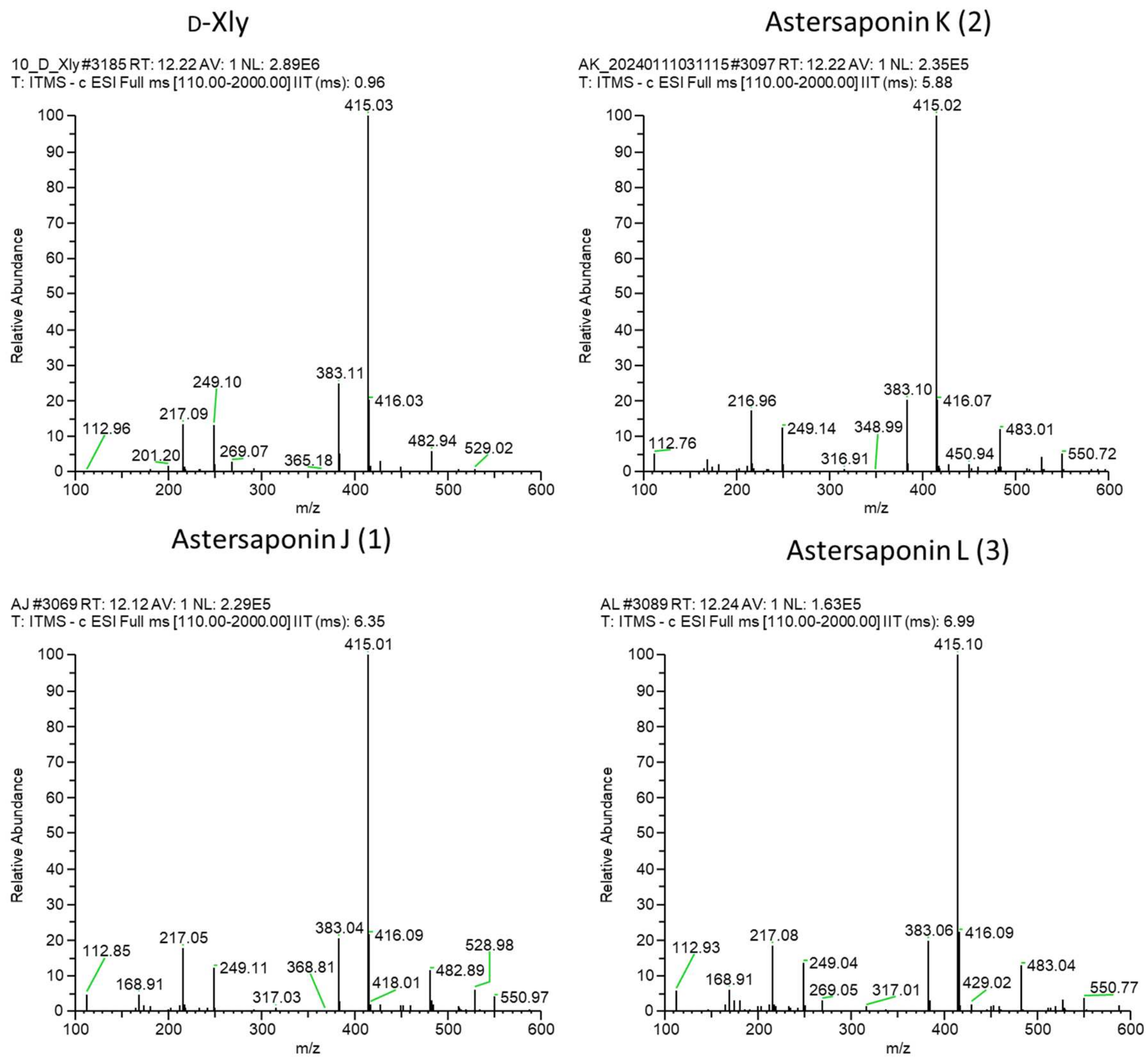

Figure S25. Full MS spectrum of derivatized xylose and hydrolyzed samples.

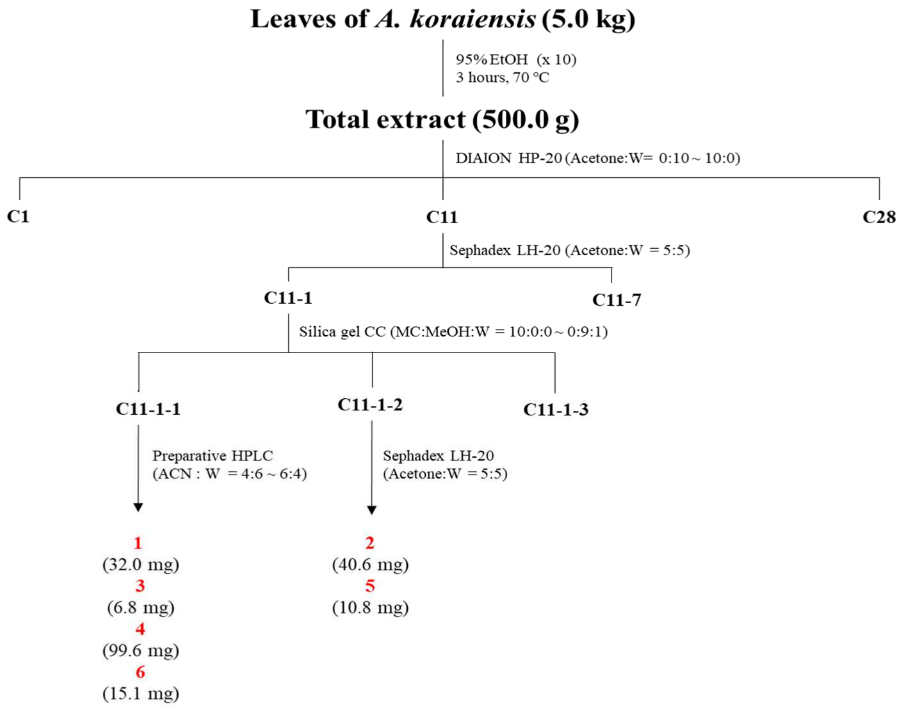

Scheme S1. Isolation scheme of compounds **1**–**6** from the 95% EtOH extract of *A*. *koraiensis* leaves

Table S1. ^{1}H and ^{13}C NMR spectroscopic data of compound **4** (δ in ppm, pyridine-d_5, 500 and 125 MHz)

Position[a]	Aglycon		Position[a]	Sugar	
	δ_H Multi (*J* in Hz)	δ_C		δ_H Multi (*J* in Hz)	δ_C
1	1.26 br m / 2.30 br m	44.2	1′	5.19 d (7.5)	105.4
2	4.77 br m	70.7	2′	4.05 br m	74.2
3	4.32 br m	82.6	3′	4.08 br m	87.5
4	—	42.7	4′	4.12 br m	69.3
5	1.71 br m	47.5	5′	3.84 br m	77.7
6	1.78 br m / 1.83 br m	17.8	6′	4.28 br m / 4.42 br m	62.0
7	1.55 br m / 1.74 br m	33.1	1″	5.23 d (7.5)	106.2
8	—	40.0	2″	4.02 br m	75.1
9	1.80 br m	47.4	3″	4.16 br m	78.1
10	—	36.8	4″	4.18 br m	70.7
11	1.80 br m / 2.05 br m	23.8	5″	3.70 br m / 4.32 br m	67.2
12	5.62 br s	123.9	1‴	6.56 br s	93.1
13	—	144.2	2‴	4.53 br m	75.2
14	—	42.0	3‴	4.60 br m	69.4
15	1.74 br m / 2.22-2.43 br m	35.9	4‴	4.43 br m	65.3
16	5.26 br s	73.9	5‴	3.92 br m / 4.47 br m	62.2
17	—	49.4	1⁗	5.70 br s	100.8
18	3.60 br m	41.1	2⁗	4.77 br m	71.5
19	1.34 br m / 2.76 br m	46.8	3⁗	4.54 br m	73.9
20	—	30.8	4⁗	4.52 br m	83.6
21	1.29 br m / 2.22-2.43 br m	35.8	5⁗	4.40 br m	68.6
22	2.14 br m / 2.26 br m	32.0	6⁗	1.74 d (6.0)	18.4
23	4.30 br d (10.5) / 3.67 br d (10.5)	64.8	1⁗′	5.39 d (7.5)	104.4
24	1.35 s	14.9	2⁗′	3.92	75.9
25	1.61 s	17.2	3⁗′	4.19	82.2
26	1.19 s	17.5	4⁗′	4.08	68.8
27	1.78 s	27.1	5⁗′	3.50 br m / 4.04 br m	67.0
28	—	175.8	1⁗″	6.27 br s	102.4
29	0.99 s	33.1	2⁗″	4.78 br m	72.3

30	1.15 s	24.6	3″″″	4.58 br m	72.4
			4″″″	4.30 br m	72.8
			5″″″	4.95 br m	69.1
			6″″″	1.64 d (6.5)	18.5
			1″″″′	5.05 d (7.0)	105.9
			2″″″′	3.94 br m	74.2
			3″″″′	3.96 br m	77.8
			4″″″′	4.04 br m	69.6
			5″″″′	3.50 br m / 4.04 br m	66.7

Table S2. ^{1}H and ^{13}C NMR spectroscopic data of compound **5** (δ in ppm, pyridine-d_5, 500 and 125 MHz)

Position[a]	Aglycon		Position[a]	Sugar	
	δ_H Multi (J in Hz)	δ_C		δ_H Multi (J in Hz)	δ_C
1	1.26 / 2.29	44.2	1′	5.16 d (8.0)	105.5
2	4.82	70.5	2′	4.04	75.4
3	4.32	83.1	3′	4.20	78.5
4	—	42.7	4′	4.20	71.4
5	1.70	47.8	5′	3.90	78.2
6	1.78 / 1.82	18.1	6′	4.32 / 4.46	62.5
7	1.54 / 1.73	33.2	1″	6.51	93.3
8	—	40.5	2″	4.53	75.3
9	1.80	47.6	3″	4.60	68.9
10	—	37.0	4″	4.43	65.5
11	1.80 / 2.05	24.0	5″	3.92 / 4.48	62.4
12	5.62	123.0	1‴	5.66	100.8
13	—	144.4	2‴	4.77	71.6
14	—	42.2	3‴	4.54 dd (9.0, 9.0)	82.3
15	1.74 / 2.23-2.45	36.2	4‴	4.51 dd (9.0, 9.0)	77.9
16	5.28	74.1	5‴	4.40	68.7
17	—	49.6	6‴	1.73 d (6.0)	18.6
18	3.38 dd (5.0, 14.0)	41.2	1‴′	5.40 d (7.0)	104.7
19	1.34 / 2.75	47.0	2‴′	3.92	76.0
20	—	30.9	3‴′	4.22	83.7
21	1.29 / 2.45-2.23	36.1	4‴′	4.08	69.5
22	2.18 / 2.27	32.1	5‴′	3.34 dd (10.5, 10.5) / 4.03-4.05	66.8
23	3.65 / 4.35	65.5	1‴″	6.20	102.6
24	1.34	15.1	2‴″	4.77	72.4
25	1.60	17.4	3‴″	4.58 dd (3.0, 8.5)	72.5
26	1.18	17.7	4‴″	4.29	74.1
27	1.77	27.2	5‴″	4.94 dq (6.0, 9.5)	69.8
28	—	176.0	6‴″	1.63 d (6.5)	18.6
29	0.99 s	33.2	1‴‴	5.08 d (7.5)	105.8

30	1.15 s	24.8	2′′′′′	3.94	75.4
			3′′′′′	3.96	78.3
			4′′′′′	4.04	70.8
			5′′′′′	3.39 dd (11.0, 11.0) / 4.03-4.05	66.9

Table S3. ^{1}H and ^{13}C NMR spectroscopic data of compound **6** (δ in ppm, pyridine-d_5, 500 and 125 MHz)

Position[a]	Aglycon		Position[a]	Sugar	
	δ_H Multi (J in Hz)	δ_C		δ_H Multi (J in Hz)	δ_C
1	1.26 / 2.29	44.2	1′	5.14 d (7.5)	105.4
2	4.82	70.7	2′	4.03	74.4
3	4.32	82.9	3′	4.07	87.6
4	—	42.8	4′	4.13	69.4
5	1.70	47.6	5′	3.84 m	77.9
6	1.78 / 1.82	18.0	6′	4.28 / 4.40	62.2
7	1.54 / 1.73	33.2	1″	5.20 d (7.5)	106.3
8	—	40.1	2″	4.00	75.3
9	1.80	47.7	3″	4.15	78.2
10	—	36.9	4″	4.16	70.9
11	1.80 / 2.05	24.0	5″	3.70 / 4.30	67.4
12	5.62	123.0	1‴	6.49 br s	93.4
13	—	144.3	2‴	4.52	75.2
14	—	42.2	3‴	4.53	69.9
15	1.74 / 2.23-2.45	36.1	4‴	4.39	66.1
16	5.28	73.9	5‴	3.95 / 4.55	63.0
17	—	49.5	1⁗	5.70 br s	101.0
18	3.38 dd (5.0, 14.0)	41.2	2⁗	4.52	71.9
19	1.34 / 2.75	47.0	3⁗	4.50	72.7
20	—	30.8	4⁗	4.33	83.9
21	1.29 / 2.45-2.23	35.9	5⁗	4.35	68.6
22	2.18 / 2.27	32.0	6⁗	1.72 d (5.5)	18.4
23	3.65 / 4.35	65.1	1⁗′	5.09 d (8.0)	106.8
24	1.34	15.0	2⁗′	4.00	76.2
25	1.60	17.3	3⁗′	4.19	83.3
26	1.18	17.6	4⁗′	4.05	69.3
27	1.77	27.1	5⁗′	3.44 dd (11.0, 11.0) / 4.18	67.3
28	—	175.9	1⁗″	6.20 d (1.0)	102.6
29	0.99 s	33.2	2⁗″	4.76 br s	72.5

30	1.15 s	24.7	3''''''	4.56	72.6
			4''''''	4.29	74.1
			5''''''	4.92	69.9
			6''''''	1.66 d (6.0)	18.6
